# Problems and solutions in quantifying cerebrovascular reactivity using BOLD-MRI

**DOI:** 10.1162/imag_a_00556

**Published:** 2025-05-02

**Authors:** Jacob B. Schulman, Kamil Uludağ

**Affiliations:** Department of Medical Biophysics, University of Toronto, Toronto, ON, Canada; Krembil Brain Institute, University Health Network, Toronto, ON, Canada; Center for Neuroscience Imaging Research, Institute for Basic Science, Suwon, Republic of Korea; Department of Biomedical Engineering, Sungkyunkwan University, Suwon, Republic of Korea; Physical Sciences, Sunnybrook Research Institute, Toronto, ON, Canada

**Keywords:** CVR, BOLD, MRI, simulations, deoxyhemoglobin

## Abstract

Cerebrovascular reactivity (CVR) imaging is used to assess the vasodilatory capacity of cerebral blood vessels. While blood flow (*CVR_CBF_*), blood velocity (*CVR_v_*), and preferably blood volume changes (*CVR_CBV_*) are used to represent physiological CVR, quantifying these measures is fraught with acquisition challenges in humans. Consequently, blood oxygenation level-dependent (BOLD)-MRI CVR (*CVR_BOLD_*) is the most widely used MRI-based CVR method, even though it arguably provides the most indirect estimation of CVR. In this paper, we sought to holistically address the quantitative capacity and shortcomings of*CVR_BOLD_*. To do so, we developed a*CVR_BOLD_*simulation framework and, together with data from the*CVR_BOLD_*literature, addressed whether and to what extent*CVR_BOLD_*accurately reflects CVR, and with which parameters*CVR_BOLD_*varies most. In short, we show the following:*CVR_BOLD_*does not necessarily correspond to physiological measures of CVR and depends on physiological (e.g., hematocrit) and acquisition (e.g., field strength) parameters;*CVR_BOLD_*is dependent on the stimulus protocol (e.g., breath-holding vs. controlled hypercapnia) chosen to elicit a vasoactive response; resting-state*CVR_BOLD_*does not necessarily reflect breath-hold*CVR_BOLD_*, likely due to confounding neuronal activity; in stenotic disease and steal physiology,*CVR_BOLD_*results from a combination of factors which do not necessarily reflect the underlying CVR. We are confident that this work will provide researchers and clinicians with invaluable insights and advance the field of cerebrovascular imaging by enabling more accurate quantification of CVR in both health and disease.

## 1 Background

The cerebral vasculature is finely regulated in the healthy brain, such that adequate blood flow is in continuous supply to the cerebral tissue. As such, in addition to responding to changes in perfusion pressure via autoregulation (Lassen, 1959) and neuronal activity via neurovascular coupling (Attwell et al., 2010), the cerebral vasculature is remarkably responsive to CO_2_, and to an extent, hypoxia (Kety & Schmidt, 1948;Mardimae et al., 2012). Specifically, it is well known that increased partial pressure of CO_2_(PCO_2_) results in transient vasodilation and increased cerebral blood flow (Kety & Schmidt, 1948). Researchers and clinicians have long exploited this phenomenon by administering vascular challenges (e.g., increased CO_2_in the inspired air) while recording the magnitude of the corresponding vascular response (Fisher & Mikulis, 2021;Sleight et al., 2021)—this practice is termed cerebrovascular reactivity (CVR) imaging.

CVR imaging is commonly used to assess the vasodilatory capacity of stenotic vessels and additional downstream vessels in steno-occlusive disease (Duffin et al., 2018;Hartkamp et al., 2017;Sleight et al., 2021;Venkatraghavan et al., 2018). CVR imaging has also been used in various other diseases, including glioma, dementia, and stroke (Fierstra et al., 2018;Krainik et al., 2005;Pillai & Zacá, 2011;Yezhuvath et al., 2012). Given the primary use case of CVR imaging, the magnitude of the vasodilatory response is most directly assessed by measuring the cerebral blood volume (*CBV*) percent change per partial pressure change in CO_2_(i.e.,$\Delta PCO_{2}$) in the vessel(s) of interest. This index ($CVR_{CBV}$) can thus be termed the**ground-truth**CVR^*^(even though other definitions are also used in the literature, as will be described):

$$CVR_{CBV}=100\;\cdot \;\left(\frac{\frac{CBV-CBV_{0}}{CBV_{0}}}{\Delta PCO_{2}}\right)$$ (1.1)

Although it is the ideal measure,$CVR_{CBV}$is seldom reported in the literature (Donahue et al., 2009;Lu et al., 2003) due to challenges of imaging changes in*CBV*. Thus, CVR is more often and easily estimated in terms of the resulting percent change in cerebral blood flow (*CBF*), and to a lesser extent, blood velocity (*v*); these physiological indices are hereafter termed$CVR_{CBF}$and$CVR_{v}$, respectively:

$$CVR_{CBF}=100\;\cdot \;\left(\frac{\frac{CBF-CBF_{0}}{CBF_{0}}}{\Delta PCO_{2}}\right)$$ (1.2)

$$CVR_{v}=100\;\cdot \;\left(\frac{\frac{v-v_{0}}{v_{0}}}{\Delta PCO_{2}}\right)$$ (1.3)

Magnetic resonance imaging (MRI) paired with a vascular challenge is commonly used for CVR imaging and quantification in the human brain (see references inFisher et al., 2018;Sleight et al., 2021). MRI-quantified CVR represents an average response across many dilating blood vessels. Various MRI-based techniques, not limited to arterial spin labeling (ASL), vascular space occupancy (VASO), blood oxygenation level-dependent (BOLD) MRI, and phase contrast (PC) angiography, have been used for CVR quantification by exploiting changes in either blood volume, flow, or velocity (figure 2 inSleight et al., 2021). For example, VASO is used to estimate percent change in*CBV*, ASL is used to estimate percent change in*CBF*, and PC angiography is used to estimate percent change in*v*and/or*CBF*in the large vessels (Donahue et al., 2009;Lu et al., 2003;Taneja et al., 2020;Zhao et al., 2021).

Each CVR method is partially indirect given the confounds of various assumptions and acquisition/analysis parameters. However, BOLD-MRI CVR (*CVR_BOLD_*), which is used to measure T_2_^*^- or T_2_-weighted MRI signal changes associated with CVR-induced blood oxygenation change, arguably provides the most indirect estimation of CVR. In addition, given the relative ease of measurement,*CVR_BOLD_*is implemented in the vast majority—roughly 77%—of CVR-MRI studies (Sleight et al., 2021). To add further complexity, CVR imaging is performed using an array of CO_2_stimulus protocols, which either require the manipulation of$\Delta PCO_{2}$or the harnessing of endogenous$\Delta PCO_{2}$fluctuations. While earlier studies focused predominantly on the former approach (e.g., breath-holding, rebreathing, and prospective end-tidal targeting), resting-state signal-based*CVR_BOLD_*is now becoming increasingly prevalent in the CVR literature (Jahanian et al., 2014;Jahanian et al., 2017;Kannurpatti et al., 2014;Liu et al., 2021;Pinto et al., 2021;Wang et al., 2016;Zang et al., 2007;Zou et al., 2008).

In this paper, we first sought to briefly describe the relationship between the different formulations of physiological CVR (as described inEqs. 1.1–1.3). We then developed and applied a novel*CVR_BOLD_*simulation framework and interrogated the*CVR_BOLD_*literature to address whether and to what extent*CVR_BOLD_*accurately reflects ground-truth CVR. First, we investigated the relationship between quantified*CVR_BOLD_*and physiological/acquisition parameters, such as the baseline blood oxygenation and magnetic field strength. Using simulations and our own experimental data, we then investigated whether different stimulus protocols, such as breath-holding and resting-state, provide comparable estimates of*CVR_BOLD_*relative to one another. Finally, we simulated example cases of vascular pathology to assess the degree to which*CVR_BOLD_*reflects ground-truth CVR in disease.

## 2 Theory

### 2.1 Physiological CVR theory and simulations

As previously described, the ground-truth (i.e., most direct) definition of CVR is$CVR_{CBV}$. However, in the MRI literature, CVR has been described on separate occasions as a*CBV*change (i.e., in VASO;Donahue et al., 2009;Lu et al., 2003), a*v*change (i.e., in PC angiography;Hartkamp et al., 2012;Miller et al., 2019), and, most commonly, a*CBF*change (Cohen & Wang, 2019;De Vis et al., 2015;Hoogeveen et al., 2024;Tancredi et al., 2012;Taneja et al., 2020;Zhou et al., 2015). In addition, CVR has been described as representing vasodilatory capacity in either the large vessels (i.e., using PC angiography;Hartkamp et al., 2012;Miller et al., 2019) or microvasculature. The appropriate definition of CVR then depends on the physiological change of interest, but one must be aware that these measures can provide different representations of vascular physiology and may consequentially provide seemingly contradictory results (seeFig. 1). Thus, great care must be taken when comparing CVR results from imaging methods that exploit different representations of CVR.

**Fig. 1.**
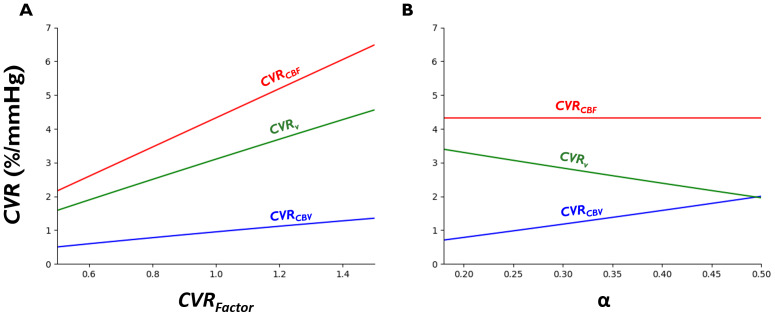
Variation among physiological CVR measures. (A) Simulated CVRs (%/mmHg) as a function of$CVR_{Factor}$. Here, CVR measures are normalized to an expected$\Delta P_{ET}CO_{2}$of ~9.25 mmHg associated with a$\Delta CBF_{p}$of 40% (Grüne et al., 2015;Poulin et al., 1996;Ramsay et al., 1993;Sato et al., 2012;Tancredi & Hoge, 2013); α = 0.29 (Ito et al., 2003). (B) Simulated CVRs as a function ofα(to represent variations in healthy vessels within the vascular network);$CVR_{Factor}$= 1.

Non-invasive*CBV*-based methods are not available in most sites and are rarely used in the literature for CVR measurements (Lu & Van Zijil, 2012;Sleight et al., 2021);*v*-based methods do not currently assess microvascular flow due to low spatial resolution in MRI studies (Hartkamp et al., 2012;Miller et al., 2019;Sur et al., 2020;Taneja et al., 2020);*CBF*-based methods do not usually measure venule/venous signal (due to T_1_decay and water exchange in the capillaries;Le et al., 2012); and may be confounded by the chosen post-labeling delay and blood delivery/transit times (Inoue et al., 2014;Xu et al., 2024). Therefore, although various MRI methods are available for estimating CVR, they are each confounded by limitations in acquisition and availability, such as differences in sensitivity to various blood vessel types. Nevertheless, it is also important to understand how the physiological measures of interest differ from one another**in the absence of acquisition-dependent limitations**; thus, we first sought to employ simulations to better understand the differences between these ideal physiological measures.

To perform the simulations, the CVR relationships described inEqs. 1.1–1.3are reformulated as a function of the change in partial pressure of expired CO_2_($\Delta P_{ET}CO_{2}$(mmHg)), the corresponding global*CBF*percent change$\left(\Delta CBF_{p}=100\;\cdot \;\left(\frac{CBF-CBF_{0}}{CBF_{0}}\right)\right)$, and a CVR heterogeneity factor ($CVR_{Factor}$). The$CVR_{Factor}$is a novel term used in this work to represent spatial heterogeneity in physiological CVR across the cerebral vasculature. The$CVR_{Factor}$represents the factor increase (> 1) or decrease (< 1) in percent flow change for a given simulated voxel relative to the global$\Delta CBF_{p}$in the brain (i.e., if$CVR_{Factor}$= 2, simulated voxel$CVR_{CBF}$corresponds to double the$\Delta CBF_{p}$, normalized to$\Delta P_{ET}CO_{2}$). Thus,$CVR_{Factor}$is effectively a proxy to voxel-specific$CVR_{CBF}$, scaled by$\Delta CBF_{p}$and$\Delta P_{ET}CO_{2}$.

$$CVR_{CBF}=\frac{\left(\Delta CBF_{p}\;\cdot \;CVR_{Factor}\right)}{\Delta P_{ET}CO_{2}}$$ (2.1)

To quantify$CVR_{CBV}$(Eq. 2.2) in terms of$\Delta CBF_{p}$, Grubb’s relationship is used (Grubb et al., 1974).$CVR_{CBV}$then depends on$\Delta CBF_{p}$, the$CVR_{Factor}$, and Grubb’s exponent (α) which governs the relationship between*CBF*and*CBV*changes as$\left(\frac{CBV}{CBV_{0}}\right)={\left(\frac{CBF}{CBF_{0}}\right)}^{\alpha }$.

$$CVR_{CBV}=100\;\cdot \;\frac{\left[{\left(1+\frac{\Delta CBF_{p}\;\cdot \;CVR_{Factor}}{100}\right)}^{\alpha }-1\right]}{\Delta P_{ET}CO_{2}}$$ (2.2)

αwas found in earlier studies to be 0.38 (Grubb et al., 1974), but more recent studies suggest it to be roughly 0.29 on average in the human brain (Ito et al., 2003) and 0.18 for the subset of vessels with deoxygenated blood (Chen & Pike, 2010). That is, for the same$\Delta CBF_{p}$, veins are expected to yield a smaller Δ*CBV*relative to capillaries/arteries. Note that this value is also likely to depend on the stimulus/challenge duration (seeUludağ & Blinder 2018).

To quantify$CVR_{v}$(Eq. 2.3) in terms of$\Delta CBF_{p}$,Eqs. 1.3and2.1–2.2were combined with the well-known*CBF*=v*CBVrelationship (here,*CBV*is %,*v*is min^-1^, and*CBF*is %/min)^†^.

$$CVR_{v}=100\;\cdot \;\frac{\left(\left(\frac{\left(\left(\frac{\Delta CBF_{p}\;\cdot \;CVR_{Factor}}{100}\right)+1\right)}{{\left(1+\frac{\Delta CBF_{p}\;\cdot \;CVR_{Factor}}{100}\right)}^{\alpha }-1}\right)-1\right)}{\Delta P_{ET}CO_{2}}$$ (2.3)

InFigure 1, vessels were simulated as a function of either the$CVR_{Factor}$or α. ForFigure 1A, the simulated vessels for each$CVR_{Factor}$can be thought of as being from different vascular territories (e.g., middle vs. posterior cerebral arteries); forFigure 1B, the simulated vessels for eachαcan be thought of as being within the same vascular path (for example, artery to capillary to vein). Given that the literature suggests thatαranges, at the very least, from ~0.18 to 0.4 (Chen & Pike, 2010;Grubb et al., 1974;Ito et al., 2003), we have simulatedαjust beyond this range inFigure 1B.

As shown inFigure 1A, the chosen definition of CVR is highly determinative of the observed response**magnitude**; as*CBF*is proportional to the product of*CBV*and*v*, the*CBF*response should be larger than its constituent parts, which is supported by the literature where average$CVR_{CBF}$in the human brain is ~3.5–7%/mmHg (Cohen & Wang, 2019;De Vis et al., 2015;Hoogeveen et al., 2024;Tancredi et al., 2012;Taneja et al., 2020;Zhou et al., 2015) and average$CVR_{CBV}$is ~0.4%/mmHg (when normalized to the expected$\Delta P_{ET}CO_{2}$during breath-holding) (Donahue et al., 2009;Lu et al., 2003). While$CVR_{v}$is similarly expected to be lower than$CVR_{CBF}$, CVR is reported in the*large vessels*when using PC angiography (~4–8%/mmHg (Miller et al., 2019;Sur et al., 2020;Taneja et al., 2020) in healthy controls) and is, therefore, not necessarily expected to be lower than the ASL-reported$CVR_{CBF}$values from the*microvasculature*. Despite differences in the absolute CVR values, relative maps (i.e., relative distribution of CVR values) are expected to agree when$CVR_{Factor}$is varied, given the linearity inFigure 1A.

The relative distribution of CVR values is dependent on the chosen physiological formulation of CVR when there are variations inα(Fig. 1B). It is known in the literature thatαis roughly 0.29 for whole brain circulation (Ito et al., 2003) but 0.18 for the subset of vessels with deoxygenated blood (Chen & Pike, 2010). Thus, it can be assumed thatαdecreases from arterial circulation to venous circulation, in keeping with the fact that arterial diameter is controlled by smooth muscle cells while venous diameter is more controlled by the intravascular pressure change generated at the arterial/capillary level. Logically,$CVR_{CBV}$,$CVR_{CBF}$, and$CVR_{v}$will be discordant when comparing vessels with differentαvalues (i.e., a voxel with a higherαvalue (arterially dominated) will yield a higher$CVR_{CBV}$and lower$CVR_{v}$than in a voxel with a lowerαvalue (venous dominated), even though$\Delta CBF_{p}$may be identical across these vessels, if for example, they are all within the same vascular path). Therefore,$CVR_{CBF}$and$CVR_{v}$will likely not adequately represent regional variations in the ground-truth (i.e.,$CVR_{CBV}$) whenαis varied.

In summary, the various physiological formulations of CVR cannot be expected to yield the same absolute values (Fig. 1A). In addition, the formulations of CVR should not yield the same relative maps in the presence of regional variations in the types of blood vessels in a voxel (Fig. 1B).

This section was dedicated to understanding how various formulations of physiological CVR (independent of MRI-associated acquisition confounds) differ from one another in both absolute and relative terms. For the remainder of this work,*CVR_BOLD_*, an index most widely utilized for CVR measurements, albeit beholden to the confounds of BOLD-MRI, will be the focus of our discussion.

### 2.2 *CVR _BOLD_* theory and simulations

BOLD-MRI is used to measure T_2_- or T_2_*-weighted signal changes resulting from regional changes in magnetic field inhomogeneity, predominantly assumed to arise from changes in paramagnetic deoxyhemoglobin (dOHb) (Bandettini et al., 1992;Kwong et al., 1992;Ogawa et al., 1990,^1992^) (note that signal changes can additionally arise from concurrent blood volume changes;Bright et al., 2014;Schulman et al., 2024;Thomas et al., 2013;Uludağ, 2010).

*CVR_BOLD_*is typically measured as a percent change in T_2_*-weighted signal normalized to$\Delta P_{ET}CO_{2}$(Fisher et al., 2018;Liu et al., 2019):

CVRBOLD,S=100 · (SMax−S0)S0ΔPETCO2 (3)

Here,$S_{0}$is the baseline BOLD signal (prior to the CO_2_challenge) and$S_{Max}$is the maximum BOLD signal in response to the CO_2_challenge.*CVR_BOLD_*is assumed to reflect CVR, particularly$CVR_{CBF}$, given that increased*CBF*leads to a decrease in deoxyhemoglobin (dOHb) in the capillaries and veins, and thus, an increase in T_2_*-weighted MRI signal (as summarized inLiu et al., 2019andUludağ et al., 2009). However, to link$CVR_{CBF}$with*CVR_BOLD_*, the contribution of various physiological and physical variables must be considered and assessed.

#### 2.2.1 Simulations: Signal model overview

Using a simulation framework, we sought to determine the influence that various parameters (e.g., echo time, field strength, blood volume) have on*CVR_BOLD_*quantification. The simulation framework in this work is adapted from a DSC signal model (Schulman et al., 2022,^2023^) informed by prior fMRI/DSC models (Kjolby et al., 2006,^2009^;Uludağ et al., 2009).

T_2_*-weighted MRI signal (S) is a summation of extravascular (*EES*) and intravascular (*IVS*) signal contributions,$S_{EES}$and$S_{IVS}$, respectively (Uludağ et al., 2009):

$$\begin{array}{l} S=\left(1-\underset{i}{\sum }(V_{i}\;\cdot \;\frac{CBV_{0}}{100}\;\cdot \;\Delta CBV_{i})\right)\;\cdot \;S_{EES}\\ \;\;\;\;\;\;\;+\phi \;\cdot \;\underset{i}{\sum }(V_{i}\;\cdot \;\frac{CBV_{0}}{100}\;\cdot \;\Delta CBV_{i}\;\cdot \;S_{IVS,i}) \end{array}$$ (4)

The index*i*denotes the specific vascular component within the voxel being simulated (capillary, venule, or arteriole; seeSection 2.2.2). Each signal contribution is scaled by the total baseline blood volume relative to the tissue volume ($CBV_{0}$(%)), each vessel’s volume fraction ($V_{i}$*,*reflecting the fraction of blood volume attributable to a given vessel type), and each vessel’s hypercapnia-induced decimal change in blood volume*($\Delta CBV_{i}$*) (seeSection 2.2.4for details). Note that we assume that the spin density factor (*ϕ*; the ratio of IVS-to-EES spin density) is equal to 1 (Lu et al., 2003;Uludağ et al., 2009). Therefore,Sin our simulations does not consider spin density effects, but this can easily be incorporated in the future by modifying*ϕ*. In addition,Sis normalized such that at an echo time of 0 ms,Sis equal to 1.

The signal components are separable as follows:

$$S_{EES}=e^{-\frac{TE}{1000}\;\cdot \;\left(R_{2,0,EES}^{*}+\underset{i}{\sum }\left(V_{i}\Delta CBV\;\cdot \;\Delta CBV_{i}\;\cdot \;R_{2,con,EES,i}^{*}\right)\right)}$$ (5.1)

$$S_{IVS}=e^{-\frac{TE}{1000}\;\cdot \;\left(R_{2,0,IVS}^{*}+R_{2,con,IVS}^{*}\right)}$$ (5.2)

$R_{2,con,EES,i}^{*}$and$R_{2,con,IVS}^{*}$(s^-1^) are the extravascular and intravascular magnetic relaxation rates induced by a susceptibility agent, respectively.$R_{2,0,EES}^{*}$and$R_{2,0,IVS}^{*}$(s^-1^) are the baseline magnetic relaxation rates without a susceptibility agent (i.e., when hemoglobin is fully saturated with oxygen). TE is the echo time (ms), and although it is simulated across a range of values inSection 3.1, TE is set to 30 ms for most of the simulations.

Table 1summarizes the extravascular and intravascular relaxation rates across field strength found in the literature (Uludağ et al., 2009;Zhao et al., 2007).

#### 2.2.2 Simulations: Baseline tissue/vascular properties

The baseline voxel parameters used in the simulations are based on previously reported physiological estimates (Lu & Ge, 2008;Uludağ et al, 2009;Vovenko, 1999) and are summarized inTable 2. However, note that inSections 3and4, these parameters are varied across a range of values to determine their effect on*CVR_BOLD_*quantification.

**Table 1.** The extravascular and intravascular relaxation rates across field strength found in the literature.

	${\text{R}}_{2,0,\text{EES}}^{*}$ ** [s ^-1^ ] **	${\text{R}}_{2,0,\text{IVS}}^{*}$ ** [s ^-1^ ] **	${\text{R}}_{2,\text{con},\text{IVS}}^{*}$ ** [s ^-1^ ] **	${\text{R}}_{2,\text{con},\text{EES},\text{i}}^{*}$ ** [s ^-1^ ] **
**1.5 T** **(** ***Hct*** **=** **44%)**	15.38	7.5	$25\;\cdot \;{\left(1-Y_{0}-\Delta Y\right)}^{2}$	*Capillary:* $1.81\;\cdot \;\left(1-Y_{0}-\Delta Y\right)$
				*Arteriole/Venule:* $2.02\;\cdot \;\left(1-Y_{0}-\Delta Y\right)$
**3 T** **(** ***Hct*** **=** **44%)**	18.03	20.7	$181\;\cdot \;{\left(1-Y_{0}-\Delta Y\right)}^{2}$	*Capillary:* $3.62\;\cdot \;\left(1-Y_{0}-\Delta Y\right)$
				*Arteriole/Venule:* $4.04\;\cdot \;\left(1-Y_{0}-\Delta Y\right)$
**4.7 T** **(** ***Hct*** **=** **44%)**	27.35	42.5	$319\;\cdot \;{\left(1-Y_{0}-\Delta Y\right)}^{2}$	*Capillary:* $5.67\;\cdot \;\left(1-Y_{0}-\Delta Y\right)$
				*Arteriole/Venule:* $6.33\;\cdot \;\left(1-Y_{0}-\Delta Y\right)$
**7 T** **(** ***Hct*** **=** **44%)**	32.6	116	$549\;\cdot \;{\left(1-Y_{0}-\Delta Y\right)}^{2}$	*Capillary:* $8.44\;\cdot \;\left(1-Y_{0}-\Delta Y\right)$
				*Arteriole/Venule:* $9.43\;\cdot \;\left(1-Y_{0}-\Delta Y\right)$
**3 T** **(** ***Hct*** **=** **21%)**	18.03	18	$103\;\cdot \;{\left(1-Y_{0}-\Delta Y\right)}^{2}$	*Capillary:* $1.72\;\cdot \;\left(1-Y_{0}-\Delta Y\right)\;$
				*Arteriole/Venule:* $1.93\;\cdot \;\left(1-Y_{0}-\Delta Y\right)$

Relaxation rate values and equations are fromZhao et al. (2007)andUludağ et al. (2009)at multiple field strengths. Hct (hematocrit) represents the percentage of blood containing red blood cells (seeSection 4.4for Hct simulations).${\text{Y}}_{0}$is the decimal baseline oxygen saturation of hemoglobin (seeTable 2for${\text{Y}}_{0}$values in each vascular compartment).ΔYis the change in oxygen saturation of hemoglobin (seeSection 2.2.3forΔYmodeling). TEs used for 1.5 T, 3 T, 4.7 T, and 7 T were 40 ms, 30 ms, 25 ms, and 20 ms, respectively.

**Table 2.** Summary of simulated tissue/vascular properties.

	**Tissue**		
	4		
$\text{CB}{\text{V}}_{0}$ **(%)**	Arteriole	Capillary	Venule
${\text{V}}_{\text{i}}$	0.2	0.4	0.4
${\text{Y}}_{0}$	0.95	0.81	0.67

The values in this table can be used for any arbitrary set of physiological assumptions (e.g., here tissue represents gray matter (GM), but$CBV_{0}$can be changed to 2% to simulate white matter (WM) as well). Note that changing baseline$CBV_{0}$affects scaling but not the qualitative results.

#### 2.2.3 Simulations: Modeling oxygenation change

Tissue blood oxygenation increases during hypercapnia (Jain et al., 2011;Kety & Schmidt, 1948;Poublanc et al., 2021). The change in oxygen saturation resulting from hypercapnia*($\Delta Y_{hc}$*) can be estimated from the well-known cerebral metabolic rate of oxygen (CMRO_2_) and*CBF*relationship (Mintun et al., 1984)—note that CMRO_2_remains relatively constant during mild/moderate hypercapnic challenges (Blockley et al., 2013;Chen & Pike, 2010;Jain et al., 2011;Vestergaard & Larsson, 2019;Zappe et al., 2008), allowing for the derivation ofEq. 6.1.$\Delta Y_{hc}$depends on the simulated vessel.$\Delta Y_{hc}$for vein/venule ($\Delta Y_{hc,v}$) depends on$\Delta CBF_{p}$, the oxygenation extraction (baseline arteriole oxygenation ($Y_{0,a}$)—baseline venule oxygenation ($Y_{0,v}$)), and the$CVR_{Factor}$in simulated tissue. To calculate$\Delta CBF_{p}$, we first set$\Delta P_{ET}CO_{2}$to 9.25 mmHg based on typical maximal changes during$\Delta P_{ET}CO_{2}$-based CVR studies (Bright & Murphy, 2013;Poublanc et al., 2015;Sleight et al., 2021). We then used literature data/equations (Grüne et al., 2015;Poulin et al., 1996;Ramsay et al., 1993;Sato et al., 2012;Tancredi & Hoge, 2013) to calculate$\Delta CBF_{p}$in response to a$\Delta P_{ET}CO_{2}$of 9.25 mmHg ($\Delta CBF_{p}$of ~40%). As previously discussed,$CVR_{Factor}$is a novel term used in this work to represent spatial heterogeneity in$CVR_{CBF}$across the cerebral vasculature (i.e., simulated voxels).

$$\Delta Y_{hc,v}=\frac{(Y_{0,a}-Y_{0,v})\;\cdot \;\left(\Delta CBF_{p}\;\cdot \;CVR_{Factor}\right)}{\left(100+\Delta CBF_{p}\;\cdot \;CVR_{Factor}\right)}$$ (6.1)

$\Delta Y_{hc}$for artery/arteriole ($\Delta Y_{hc,a}$) is set directly to 0, given that in healthy subjects there is negligible oxygen exchange in the arterial/arteriolar segment of the vascular network (Jain et al., 2011;Vu et al., 2023).

$$\Delta Y_{hc,a}=0$$ (6.2)

$\Delta Y_{hc}$for capillary ($\Delta Y_{hc,c}$) is half of that in simulated venule (based on the fact that the oxygen extraction in capillary (relative to arteriole) is assumed to be approximately half of that in venule (relative to arteriole)).

$$\Delta Y_{hc,c}=\frac{1}{2}\cdot \;\Delta Y_{hc,v}$$ (6.3)

The total change in oxygen saturation (ΔY) for a given vessel can then be described as the summation of its$\Delta Y_{hc}$and the oxygenation change resulting from hypoxia ($\Delta Y_{hyp}$).

$$\Delta Y=\Delta Y_{hc}+\Delta Y_{hyp}$$ (7)

$\Delta Y_{hyp}$is assumed to be 0 for all simulations, except for breath-holding (seeSection 5).

#### 2.2.4 Simulations: Modeling vasodilation

As previously mentioned (Eq. 4), hypercapnia-induced vasodilation (ΔCBV) must be included in the CVR modeling (Kety & Schmidt, 1948;Liu et al., 2019).ΔCBVdepends on the$\Delta CBF_{p}$,$CVR_{Factor}$, and Grubb’s exponent (α) (Grubb et al., 1974) in simulated tissue:

$$\Delta CBV={\left(1+\frac{\Delta CBF_{p}\;\cdot \;CVR_{Factor}}{100}\right)}^{\alpha }$$ (8)

As previously noted,αfor whole brain has been found to be roughly 0.29 (Ito et al., 2003) but 0.18 for the subset of vessels with deoxygenated blood (Chen & Pike, 2010). Thus, for our simulations we setαto 0.18 for venules, 0.29 for capillaries, and extrapolated to 0.4 for arterioles. Note thatΔCBVis relative (e.g., ifΔCBV= 1.08 (i.e., an 8% change) and$CBV_{0}$= 4% (typical values for gray matter),*CBV*would increase to 4.32%).

#### 2.2.5 Simulations: Calculation of $\text{CV}{\text{R}}_{\text{BOLD}}$

According to a recent systematic review (Sleight et al., 2021)*,*the vast majority of papers report*CVR_BOLD_*as percent signal change normalized to$\Delta P_{ET}CO_{2}$($CVR_{BOLD,S}$), described inEq. 3, whereas only 0.4% of studies report*CVR_BOLD_*as a relaxation rate change normalized to$\Delta P_{ET}CO_{2}$($CVR_{BOLD,R}$):

$$\Delta R_{2}^{*}=-\left(\frac{1}{TE}\right)\;\cdot \;ln\left(\frac{S_{max}}{S_{0}}\right)$$ (9.1)

$$CVR_{BOLD,R}=\frac{\left|\Delta R_{2}^{*}\right|}{\Delta P_{ET}CO_{2}}$$ (9.2)

As a reminder,$S_{0}$is the baseline signal (when$\Delta CBF_{p}$is set to 0),$S_{Max}$is the maximum signal (e.g., when$\Delta CBF_{p}$is set to 40%), and$\Delta R_{2}^{*}$is the corresponding change in relaxation rate. The preponderance of$CVR_{BOLD,S}$relative to$CVR_{BOLD,R}$is very surprising given the well-known relationship between echo time and T_2_* signal change (Eqs. 5.1and5.2). That is, higher TE yields higher calculated values of$CVR_{BOLD,S}$(e.g., see figure 1 inHavlicek et al., 2017), whereas$CVR_{BOLD,R}$should be (almost) TE independent. As previously mentioned,$\Delta P_{ET}CO_{2}$is set to 9.25 mmHg for these simulations.

## 3 *CVR _BOLD_* Dependency on Acquisition Parameters

### 3.1 Echo time

**Fig. 2.**
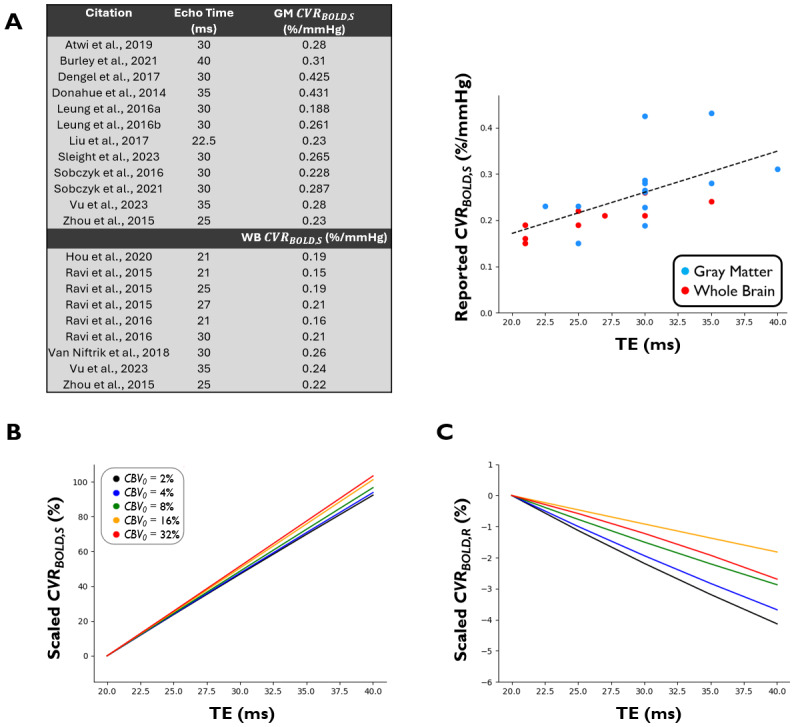
Relationship between echo time and$CVR_{BOLD,S}$estimation. (A)**Literature:**Table and plot of reported$CVR_{BOLD,S}$values (%/mmHg) in average GM and WB across a range of echo times (ms). (B)$CVR_{BOLD\text{,}S}$**Simulations:**Relationship between$CVR_{BOLD,S}$and echo time. (C)$CVR_{BOLD\text{,}R}$**Simulations:**Relationship between$CVR_{BOLD,R}$and echo time. Note that the y-axis range is much smaller relative to B.*CBV_0_*was varied from 2% to 32%. Note that scaled*CVR_BOLD_*represents the percent change of*CVR_BOLD_*in relation to*CVR_BOLD_*at 20 ms (i.e.,$\text{Scaled }CVR_{BOLD\text{,}R}=\frac{100\;\cdot \;(CVR_{BOLD\text{,}R}(TR)-CVR_{BOLD\text{,}R}(TE=20\;ms))}{CVR_{BOLD\text{,}R}(TE=20\;ms)}$).

In addition to investigating the effect of TE on*CVR_BOLD_*using our simulations, we surveyed gray matter (GM) and whole brain (WB)$CVR_{BOLD,S}$values reported in the literature across a range of TEs at 3 T (Fig. 2).

Literature values at 3 T (Atwi et al., 2019;Burley et al., 2021;Dengel et al., 2017;Donahue et al., 2014;Hou et al., 2020;Leung, Duffin, et al., 2016;Leung, Kim, et al., 2016;Liu, Welch, et al., 2017;Ravi et al., 2015,^2016^;Sleight et al., 2023;Sobczyk et al., 2016,^2021^;Triantafyllou et al., 2011;Van Niftrik et al., 2018;Vu et al., 2023;Zhou et al., 2015) suggest that a positive, linear TE-dependency exists when reporting as$CVR_{BOLD,S}$(Fig. 2A), in agreement with our simulations (Fig. 2B). In fact, Triantafyllou et al. specifically investigated the dependency of$CVR_{BOLD,S}$on TE and observed a strong positive linear correlation (Triantafyllou et al., 2011). Note that this relationship assumes that signal is above the noise floor (i.e., we would not necessarily expect this linear relationship to hold true in regions where baseline signal is low and dominated by noise). While there are insufficient data for$CVR_{BOLD,R}$across a range of TEs in the literature, our simulations show that the TE-dependency is largely eliminated when CVR is calculated as$CVR_{BOLD,R}$(i.e.,$CVR_{BOLD,R}$values differ by only ~2–4% between TE = 20 ms and TE = 40 ms) (Fig. 2C).

Given these insights, we recommend that researchers and clinicians implementEqs. 9.1–9.2for*CVR_BOLD,R_*calculation, to reduce the TE-dependency associated with$CVR_{BOLD,S}$, allowing for an easier comparison of*CVR_BOLD_*values across studies.

### 3.2 Field strength

An increased contrast-to-noise ratio makes a compelling case for the implementation of increased magnetic field strength in*CVR_BOLD_*studies; however, the relationship between field strength and both$CVR_{BOLD,S}$and$CVR_{BOLD,R}$must be considered.

**Fig. 3.**
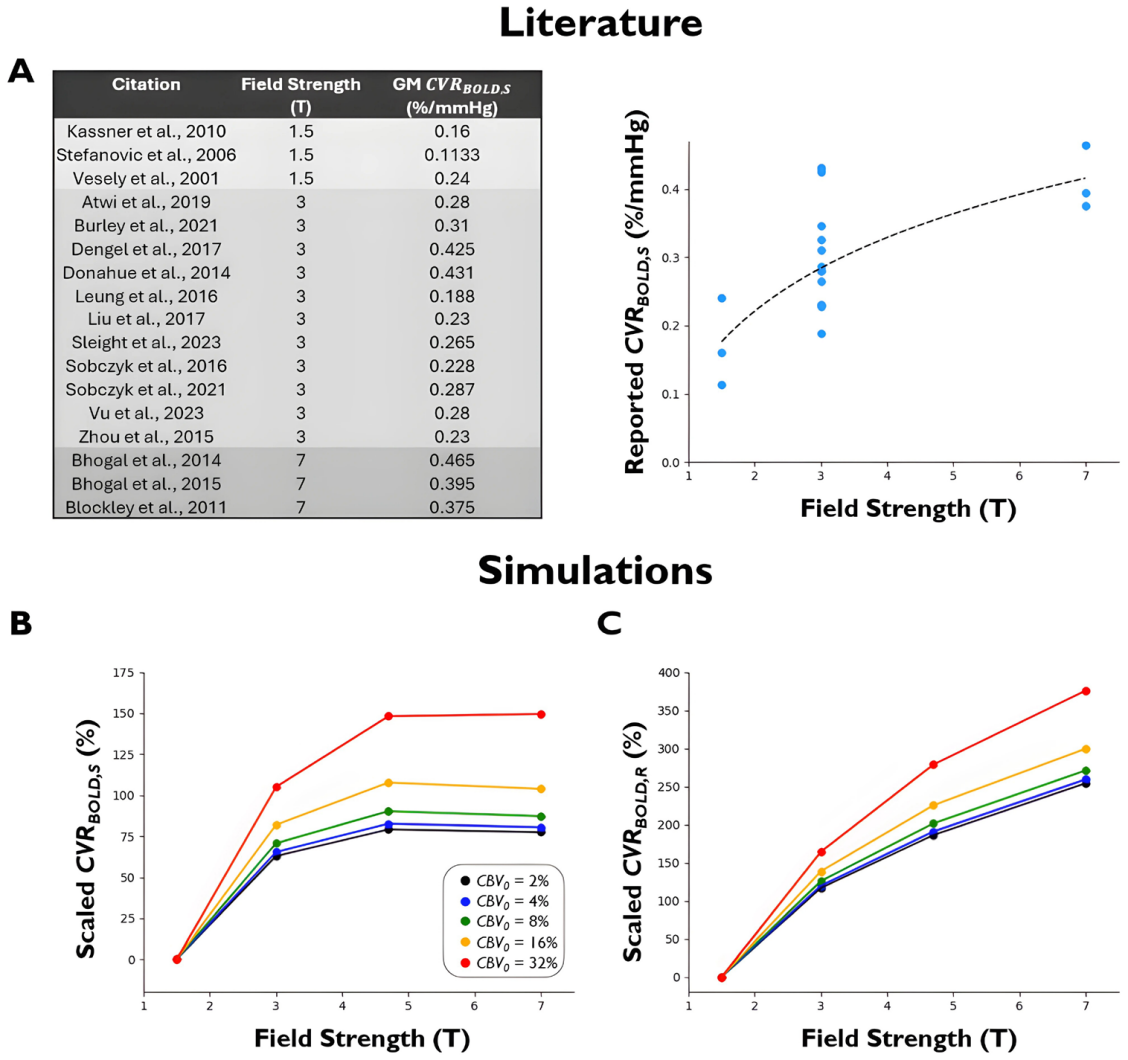
Relationship between field strength and*CVR_BOLD_*estimation. (A)**Literature:**Table and plot of reported$CVR_{BOLD,S}$values (%/mmHg) in average GM across a range of field strengths. (B)$CVR_{BOLD\text{,}S}$**Simulations:**Relationship between$CVR_{BOLD,S}$and field strength. (C)$CVR_{BOLD\text{,}R}$**Simulations:**Relationship between$CVR_{BOLD,R}$and field strength.*CBV_0_*was varied from 2 to 32%. Note that scaled*CVR_BOLD_*represents the percent change of*CVR_BOLD_*in relation to*CVR_BOLD_*at 1.5 T (i.e.,$\text{Scaled }CVR_{BOLD,R}=\frac{100\;\cdot \;\left(CVR_{BOLD,R}\left(B_{0}\right)-CVR_{BOLD,R}\left(B_{0}=1.5\text{T}\right)\right)}{CVR_{BOLD,R}\left(B_{0}=1.5\text{T}\right)}$).

Triantafyllou et al. investigated$CVR_{BOLD,S}$in GM at 1.5 T and 3 T, reporting an approximate 1.76-fold increase in percent signal change normalized to flow change as field strength doubled (Triantafyllou et al., 2011). In a breath-holding experiment, Peng et al. found a 1.66-fold increase in percent signal change as field strength doubled from 1.5 T to 3 T (Peng et al., 2020). Driver et al. investigated$CVR_{BOLD,R}$in GM at 3 T and 7 T, reporting an approximate doubling in relaxation rate change as field strength increased by 2.33-fold (Driver et al., 2010). Note that in comparison to*CVR_BOLD_*, ASL-derived CVR (*CVR_ASL_*), as expected, does not significantly depend on echo time or field strength (Nöth et al., 2006). As these studies only compared results across two field strengths, it is difficult to identify whether the field strength relationship is linear across a wider range. Therefore, we used our simulations to determine the relationship between field strength (simulated across 1.5 T, 3 T, 4.7 T, and 7 T; seeTable 1) and quantified$CVR_{BOLD,S}$and$CVR_{BOLD,R}$(Fig. 3).

GM$CVR_{BOLD,S}$values reported in the literature (Atwi et al., 2019;Bhogal et al., 2014;Bhogal et al., 2015;Blockley et al., 2011;Burley et al., 2021;Dengel et al., 2017;Donahue et al., 2014;Kassner et al., 2010;Leung, Duffin, et al., 2016;Leung, Kim, et al., 2016;Liu, Welch, et al., 2017;Sleight et al., 2023;Sobczyk et al., 2016,^2021^;Stefanovic et al., 2006;Vesely et al. 2001;Vu et al., 2023;Zhou et al., 2015) empirically demonstrate a logarithmic relationship (R^2^= 0.54) with field strength (Fig. 3A), although more data are likely needed to verify this relationship.

Like the aforementioned literature values, the simulations show that*CVR_BOLD_*does not increase linearly, but rather in a quasi-logarithmic manner. In addition to a global scaling effect, simulated voxels with higher*CBV*yield disproportionately larger increases in$CVR_{BOLD,R}$and$CVR_{BOLD,S}$as a function of field strength—in theory, this would likely result in regional scaling differences between*CVR_BOLD_*maps of different field strengths in regions containing large veins/arteries. However, in comparing regions with low blood volume (i.e., majority of the GM and WM), regional scaling differences are minimal.

Given the relationship’s deviation from linearity, a non-linear correction factor can be implemented to remove the influence of field strength on*CVR_BOLD_*measures. Upon fitting the$CVR_{BOLD,R}$and$CVR_{BOLD,S}$simulations, we found that logarithmic-radical fits best corrected for$CVR_{BOLD,R}$(R^2^= 0.999) and$CVR_{BOLD,S}$(R^2^= 0.99):

$$CVR_{BOLD,R,Scaled}=\frac{CVR_{BOLD,R}}{2.02\;\cdot \;{\left(\text{ln}(B_{0}\left(T\right))\right)}^{0.79}}$$ (10.1)

$$CVR_{BOLD,S,Scaled}=\frac{CVR_{BOLD,S}}{3.11\;\cdot \;\text{ln}\left(B_{0}\left(T\right)\right)\;\cdot \;{\left(B_{0}\left(T\right)\right)}^{-0.65}}$$ (10.2)

Therefore, for inter-field strength comparisons of CVR values obtained from similar populations, we recommend that a field-dependent correction factor, likeEqs. 10.1–10.2, is utilized prior to reporting*CVR_BOLD_*values to ensure better inter-field strength comparability. Ideally, this correction factor would be derived and validated empirically by collecting*CVR_BOLD_*data on the same subjects at multiple magnetic field strengths and then compared with the relationship in the simulations.

**Note that for simplicity, we only quantify*****CVR_BOLD_*****as$\text{CV}{\text{R}}_{\text{BOLD},\text{R}}$for the remainder of this work.**Although not explicitly shown,$CVR_{BOLD,S}$results are qualitatively identical to$CVR_{BOLD,R}$in the forthcoming relationships.

## 4 *CVR _BOLD_* Dependency on Physiological Parameters

The quantitative relationships between*CVR_BOLD_*and various tissue/vascular properties, such as the hematocrit (*Hct*) and arteriolar blood volume, remain understudied. We explored the literature and used our simulations to understand the dependency of*CVR_BOLD_*on a comprehensive set of physiological parameters.

### 4.1 Baseline blood volume

One parameter that is expected to influence*CVR_BOLD_*is the underlying baseline*CBV*(*CBV_0_*), in agreement with the literature (Blockley et al., 2013;Buxton et al., 2004;Davis et al., 1998;Yablonskiy & Haacke 1994), where the relationship between*CBV_0_*and the percent BOLD change (and/or$\Delta R_{2}^{*}$) has been shown to be linear (specifically, see Eq. 1 inDavis et al., 1998and Eq. 9 inBuxton et al., 2004); this is because the change in the absolute amount of dOHb (susceptibility agent) in a voxel during hypercapnia is scaled by*CBV_0_*, which ultimately scales the observed BOLD signal change. Thus, according to this relationship derived from these standard BOLD signal models (Buxton et al., 2004;Davis et al., 1998), doubling*CBV_0_*results in a doubling of the measured BOLD-MRI signal change, and thus,*CVR_BOLD_*. Consequently, comparing*CVR_BOLD,GM_*with*CVR_BOLD,WM_*, for example, is heavily biased by the roughly doubled*CBV_0_*in GM (*CBV_0_*~ 4%) relative to WM (*CBV_0_*~ 2%). Therefore, making the claim that “*CVR_BOLD_*reflects$CVR_{CBF}$” is just as valid as claiming that “*CVR_BOLD_*reflects*CBV_0,_*” which poses a considerable limitation on*CVR_BOLD_*in its current state. To clarify, if*CVR_BOLD_*reflected*CVR_CBV_*(that is, the change in*CBV*), this would be a benefit, not a limitation. However,*CVR_BOLD_*scales linearly with**baseline*****CBV*****(*****CBV_0_*****)**. This is a limitation as it confounds the reactivity measure of interest with a baseline measure that is not reflective of the percent*CBV*change in response to a vascular stimulus.

In agreement with the theory (Blockley et al., 2013;Buxton et al., 2004;Davis et al., 1998;Yablonskiy & Haacke 1994),*CVR_BOLD,GM_*has empirically been found to be roughly double*CVR_BOLD,WM_*in the literature, while*CVR_ASL,GM_*has been found to be roughly the same as*CVR_ASL,WM_*(Taneja et al., 2020;Xu et al., 2024). Since DSC-MRI calculated*CBV_0_*(Ostergaard, 2005;Schulman et al., 2023) and*CVR_BOLD_*both scale roughly linearly with*CBV_0_*, dividing*CVR_BOLD_*by*CBV_0_*(for example, measured separately using DSC) is a promising solution for removing the confound of*CBV_0_*from measures of*CVR_BOLD_*. The DSC data can be acquired using Gd-based contrast (in clinical scenarios, this is often routinely administered for glioma and neurovascular disease) or dOHb-based contrast (administered using sequential gas delivery systems, as is used in many CVR studies to induce hypercapnia). This novel approach could then be validated by comparing with*CVR_ASL_*which is assumed to be independent of*CBV_0_*(Petersen et al., 2006;Taneja et al., 2020).

### 4.2 Arteriolar blood volume

**Fig. 4.**
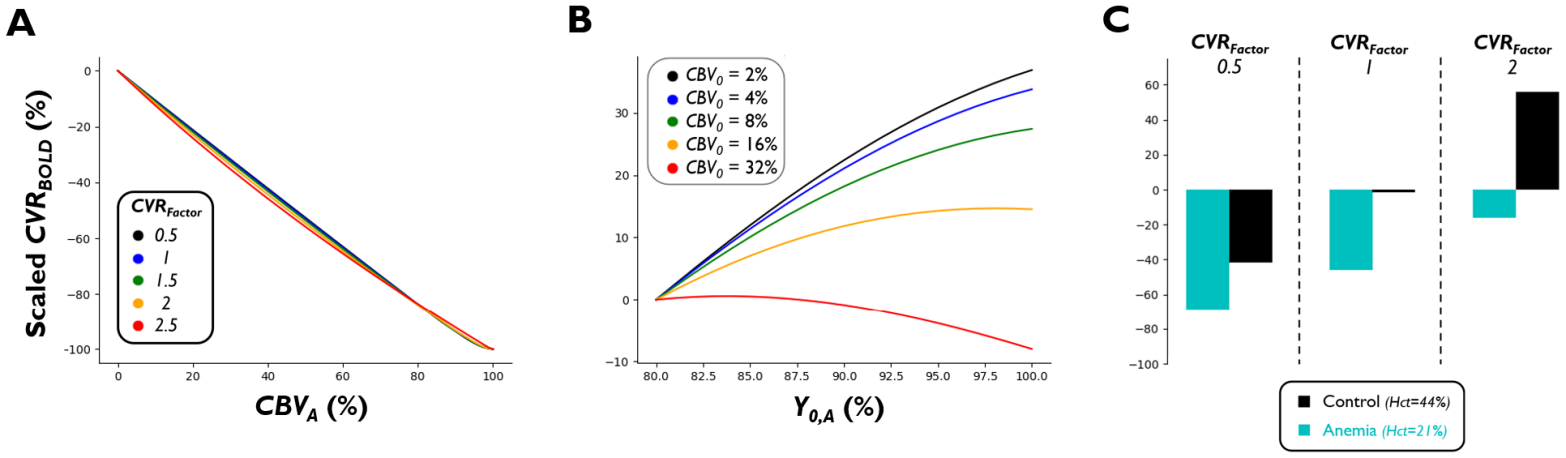
Simulated relationship between*CVR_BOLD_*and blood/tissue parameters. Scaled*CVR_BOLD_*represents the percent change of*CVR_BOLD_*in relation to*CVR_BOLD_*at the first datum point for subplots A–B (e.g., in (A):$ScaledCVR_{BOLD}$$=\frac{100\;\cdot \;\left(CVR_{BOLD,R}\left(CBV_{A}\right)-CVR_{BOLD,R}\left(CBV_{A}=0\right)\right)}{CVR_{BOLD,R}\left(CBV_{A}=0\right)}$). (A) Relationship between relative*CBV_A_*(percentage of*CBV0*that is arteriolar (like arteriolar V_i_inSection 2.2.2, but as a percentage)) and estimated*CVR_BOLD_*. The$CVR_{Factor}$was varied from 0.5 to 2.5 for subplot A. (B) Relationship between the baseline arteriolar blood oxygenation (${\text{Y}}_{0,\text{A}}$) and estimated*CVR_BOLD_*.*CBV_0_*was varied from 2 to 32%. (C) Relationship between the Hct (%) and estimated*CVR_BOLD_*. Black represents control (Hct = 44%) and cyan represents anemia (Hct = 21%). Scaled*CVR_BOLD_*represents the percentage difference of*CVR_BOLD_*in relation to*CVR_BOLD_*for control, when*CVR_Factor_*= 1.

The observed tissue relaxation rate change in*CVR_BOLD_*is a result of increased blood flow, reduced oxygen extraction, and ultimately, a lower proportion of deoxygenated hemoglobin in the capillaries and veins. In arterial/arteriolar blood where oxygenation is near full saturation (Severinghaus, 1979) and where oxygen does not generally exchange with tissue, oxygenation-induced relaxation rate change is negligeable. We used our simulations to demonstrate the effect of the simulated proportion of arteriolar*CBV_0_*(*CBV_A_*(%)) on the measured*CVR_BOLD_*(Fig. 4A).

While$CVR_{CBF}$and$CVR_{CBV}$are held constant,*CVR_BOLD_*reduces drastically as the simulated tissue’s vessels become arterially dominated (theoretically, there is no transverse relaxation rate change when the voxel only contains arterial/arteriolar blood). Therefore, voxels with a low*CVR_BOLD_*may have high vasodilatory capacity and only appear to have a low CVR due to a higher proportion of fully oxygenated blood. In other words,*CVR_BOLD_*has a low sensitivity to CVR in arteries/arterioles (seeSchulman et al., 2024, which shows how*CVR_BOLD_*in arteries can even be negative due to the displacement of cerebrospinal fluid). There is no straightforward way to mitigate this shortcoming, but studies should acknowledge the lack of oxygenation-induced arterial/arteriolar relaxation change as a limitation of*CVR_BOLD_*.

### 4.3 Baseline blood oxygenation

While baseline arterial/arteriolar blood oxygenation ($Y_{0,A}$(%)) is usually ≥ 97% in humans (Chan et al., 2013), it can be markedly lower (within the range of 80–90%) in certain patient populations, such as those with pulmonary disease (Echevarria et al., 2021). Thus, we sought to investigate the influence of$Y_{0,A}$on*CVR_BOLD_*(Fig. 4B). Of note, it is known that reducing$Y_{0,A}$beyond roughly 80% (i.e., ~50 mmHg) results in a hypoxia-driven cerebrovascular response (Mardimae et al. 2012); therefore, we constrained the$Y_{0,A}$parameter to avoid simulating this additional hypoxia-mediated increase in*CBF*.

The relationship between$Y_{0,A}$and*CVR_BOLD_*is heavily dependent on the*CBV_0_*of the simulated voxel (i.e.,*CVR_BOLD_*magnitude increases non-linearly with$Y_{0,A}$in tissue (*CBV_0_~*2–8%) but decreases non-linearly with$Y_{0,A}$in highly vascular voxels (e.g.,*CBV_0_*of 32%)). The observation for the highly vascular voxels is in agreement with our previous DSC MRI work, which found that∫ΔBOLD(t)induced by susceptibility agent was negatively correlated with$Y_{0,A}$in voxels with larger vessels (Schulman et al., 2023), owed to the quadratic dependency of the intravascular relaxation rate on blood oxygenation. However, the observed*CVR_BOLD_*increase as a function of$Y_{0,A}$in tissue is owed to a larger overall**extravascular**relaxation rate change magnitude at higher$Y_{0,A}$as a result of concurrent ∆*CBV*-mediated relaxation rate change (opposite in sign to the CVR-mediated relaxation rate change), which is larger in magnitude at lower$Y_{0,A}$(refer toSchulman et al., 2024). In general, these simulations reveal a complex relationship between the measured*CVR_BOLD_*and$Y_{0,A}$which is important for consideration in hypoxemic patients. In future work, incorporating the measured$Y_{0,A}$(estimated from a patient’s S_p_O_2_) into tissue*CVR_BOLD_*modeling may bypass this dependency in patients with abnormally low$Y_{0,A}$values.

### 4.4 Hematocrit

While*Hct*is measured to be around 40–54% for men and 36–48% for women (Billett, 1990),*Hct*can be as low as ~20% in anemic patients (Afzali-Hashemi et al., 2021;Kuwabara et al., 2002;Nur et al., 2009). While the literature suggests that certain forms of anemia may result in reduced*CVR_BOLD_*(Kosinski et al., 2017), it is unclear to what extent*Hct*attenuates*CVR_BOLD_*when$CVR_{CBF}$and$CVR_{CBV}$are unchanged. To examine this relationship, we simulated (seeSection 2.2.1andTable 1)*CVR_BOLD_*for control (*Hct*= 44%) and anemic (*Hct*= 21%) tissue/blood (Fig. 4C). For these simulations, we assumed that reduced*Hct*yields a disproportionally large increase in baseline blood flow, resulting in halving of the baseline*OEF*(as observed inVu et al., 2017). Although not shown, simulating a doubling of the baseline*OEF*or maintenance of the baseline*OEF*yields the same pattern observed inFigure 4Crelative to control, albeit with different amplitudes.

According to the simulations (Fig. 4C), even in the absence of any changes to the underlying CVR (i.e.,$CVR_{Factor}$= 1), the quantified*CVR_BOLD_*is reduced in simulated anemia by ~40%, relative to normal tissue—this appears regardless of whether the$CVR_{Factor}$is low (0.5) or high (2). These results have important implications when assessing*CVR_BOLD_*in anemic patients, and even when comparing results across sex—namely, while reduced$CVR_{CBF}$might partially account for the observed*CVR_BOLD_*reduction in anemia relative to controls (Afzali-Hashemi et al., 2021;Kosinski et al., 2017;Kuwabara et al., 2002;Nur et al., 2009;Sayin et al., 2022) and in women relative to men (Kassner et al., 2010), the simulations indicate that*CVR_BOLD_*increases as a function of*Hct*even when physiological CVR (i.e.,$CVR_{CBV}$or$CVR_{CBF}$) is unchanged. Upon validating this dependency experimentally (i.e., observing*CVR_BOLD_*as a function of*Hct*in healthy subjects) and simulating across a larger range of*Hct*values, incorporating an individual’s*Hct*into a*Hct*-based correction factor may allow for the correction of this*CVR_BOLD_*dependency.

Note that variations in baseline CMRO_2_would theoretically confound the relationship between*Hct*and*CVR_BOLD_*as it would alter$Y_{0}$(i.e., increased baseline CMRO_2_would yield a decrease in$Y_{0}$)*. CVR_BOLD_*is dependent on$Y_{0}$, as we have shown above (Fig. 4B).

## 5 *CVR _BOLD_* Dependency on Stimulus Design

### 5.1 Controlled hypercapnia versus breath-holding

Breath-holding is a well-accepted method in*CVR_BOLD_*studies (Bright & Murphy, 2013;Dlamini et al., 2018;Fierstra et al., 2013;Peng et al., 2020;Ratnatunga & Adiseshiah, 1990); however, there are notable limitations. One of the major limitations of breath-holding in comparison with controlled hypercapnia methods (i.e., dynamic end-tidal forcing or prospective end-tidal targeting) is the confounding effect of concurrent hypoxia (Tancredi & Hoge, 2013) whose level is dependent on the breath-hold duration (Sasse et al., 1996). To date, it is unclear how the degree of hypoxia propagates error into the quantified*CVR_BOLD_*values.

**Fig. 5.**
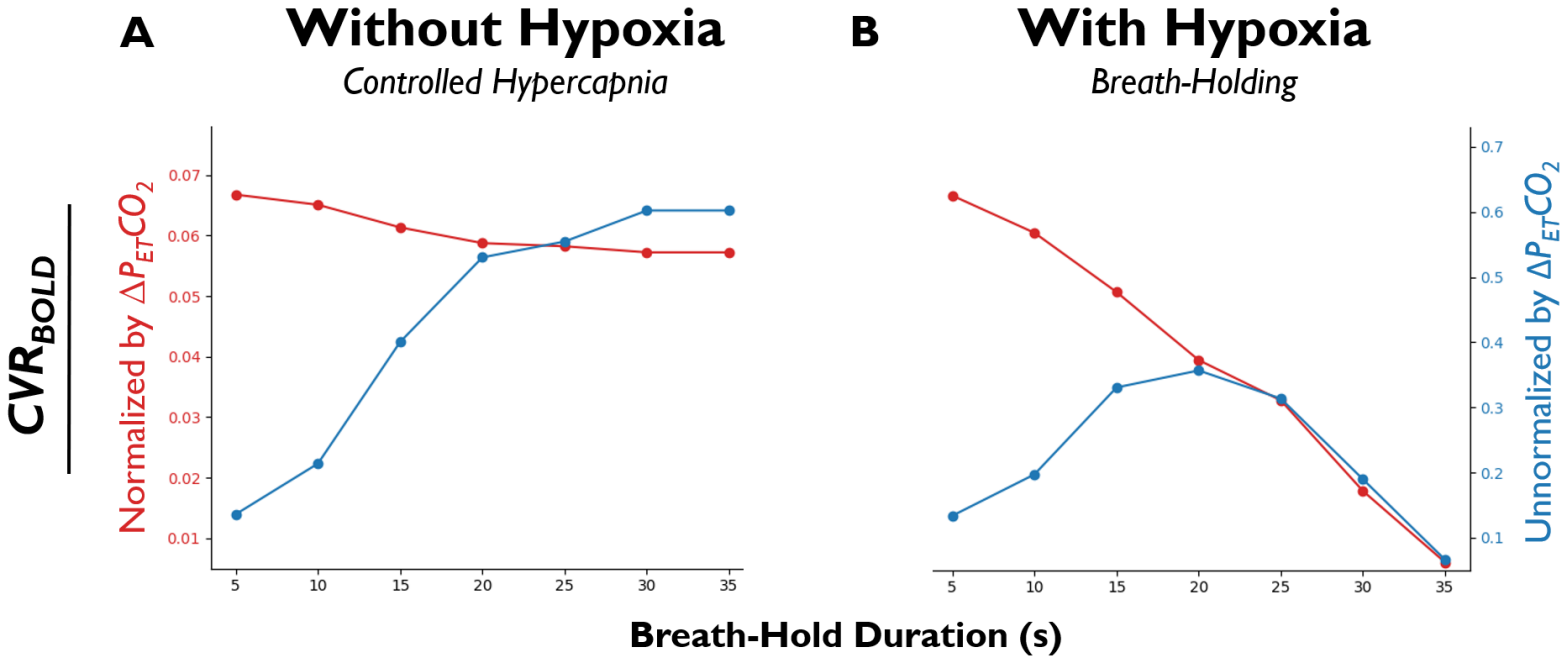
Simulated relationship between*CVR_BOLD_*estimation and breath-hold duration.*CVR_BOLD_*normalized (red; s^-1^/mmHg) or not normalized (blue; s^-1^) to$\Delta P_{ET}CO_{2}$(Supplementary Table 1andSupplementary Fig. 1). For quantification, seeEq. 9.2andSupplementary Table 1for corresponding$\Delta P_{ET}CO_{2}$values. (A) Simulated without hypoxia (to mimic controlled hypercapnia). (B) Simulated with hypoxia (to mimic breath-holding).

Using hypoxia and hypercapnia values reported in previous works (refer toSupplementary Fig. 1andSupplementary Table 1), we simulated*CVR_BOLD_*as a function of the breath-hold duration. To reiterate, breath-holding (Fig. 5B) is associated with hypoxia whereas controlled hypercapnia (Fig. 5A) is not (here, breath-hold duration is just a proxy to the$\Delta P_{ET}CO_{2}$stimulus magnitude; seeSupplementary Table 1). In addition, while some researchers (Bright & Murphy, 2013;Golestani et al., 2016;Lipp et al., 2015) normalize breath-hold-acquired*CVR_BOLD_*values to$\Delta P_{ET}CO_{2}$(red), others do not (blue) (Cohen & Wang, 2019;Jahanian et al., 2017). Thus, we investigated the quantitative consequences of these different analysis and hypercapnic stimulus design choices.

In the controlled hypercapnia simulations,$\Delta P_{ET}CO_{2}$-normalized*CVR_BOLD_*progressively decreases, though minimally, and$\Delta P_{ET}CO_{2}$-unnormalized*CVR_BOLD_*progressively increases with the stimulus magnitude until$\Delta P_{ET}CO_{2}$saturates after changing by ~10.5 mmHg (Sasse et al., 1996) (Fig. 5A). The mild decrease in normalized*CVR_BOLD_*is initially perplexing, especially given that$CVR_{CBF}$mildly increases as a function of$\Delta P_{ET}CO_{2}$(Supplementary Table 2). This becomes clearer when recognizing that BOLD begins to saturate with progressively larger increases in$\Delta P_{ET}CO_{2}$and$\Delta CBF_{p}$, as has been observed in the literature (Bhogal et al., 2014,^2015^;Duffin et al., 2017;Hoge et al., 1999) and in agreement withEq. 6.1.

In the breath-holding simulations,$\Delta P_{ET}CO_{2}$-normalized*CVR_BOLD_*decreases substantially, and$\Delta P_{ET}CO_{2}$-unnormalized*CVR_BOLD_*increases with breath-hold durations up to 20 s and then decreases as breath-hold duration increases further (Fig. 5B). This counterintuitive decline is due to a competing hypoxic effect (i.e., increased paramagnetic dOHb during hypoxia results in a positive relaxation rate change (Poublanc et al., 2021;Sayin et al., 2023;Schulman et al., 2023;Vu et al., 2021), which counteracts the negative relaxation rate change from CVR); the summation is then either an increase or decrease in relaxation, depending on the larger contributor (seeSupplementary Fig. 2). The finding that$\Delta P_{ET}CO_{2}$-unnormalized*CVR_BOLD_*values are more consistent at moderate breath-hold durations is observed in previous work, where 10, 15, and 20 s breath-hold durations were compared for*CVR_BOLD_*measurement (Bright & Murphy, 2013); here,*CVR_BOLD_*values were found to be relatively similar between 15 s and 20 s (difference of ~0–10%) as opposed to between 10 s and 15 s (difference of ~50–60%), similar to our simulated findings.

Note that studies have reported different$\Delta P_{ET}CO_{2}$values associated with breath-hold durations (e.g.,Sasse et al., 1996vs.Bright & Murphy, 2013). For this paper, we opted to implement values fromSasse et al. (1996)as they span a large range of breath-hold durations and were collected from arterial blood. However, if we input the$\Delta P_{ET}CO_{2}$values measured fromBright and Murphy (2013)into our model (instead of those fromSasse et al., 1996), we obtain$\Delta P_{ET}CO_{2}$-normalized*CVR_BOLD_*values that are much more stable across breath-hold duration (like inBright & Murphy, 2013): ~0.057, 0.055, and 0.051 s^-1^/mmHg for 10, 15, and 20 s breath-holds, respectively.

Ultimately, breath-holding is expected to yield an underestimation of*CVR_BOLD_*(normalized and unnormalized), particularly for longer breath-hold durations, in comparison with controlled hypercapnia due to the associated hypoxic effect (Supplementary Table 1). This is in keeping with the literature where*CVR_BOLD_*values in GM tend to be ~16% lower, on average, for breath-holding versus controlled hypercapnia (Atwi et al., 2019;Bright & Murphy, 2013;Dengel et al., 2017;Donahue et al., 2014;Hou et al., 2020;Leung, Duffin, et al., 2016;Leung, Kim, et al., 2016;Lipp et al., 2015;Liu, Welch, et al., 2017;Moia et al., 2020,^2021^;Murphy et al., 2011;Pinto et al., 2016;Ravi et al., 2015;Sleight et al., 2023;Sobczyk et al., 2016;Van Niftrik et al., 2018;Zhou et al., 2015). Additionally, based on the simulations, breath-hold durations of ~20–25 s maximize the contrast-to-noise ratio in breath-hold*CVR_BOLD_*experiments. However, the simulation results suggest that those seeking to limit the hypoxic effect (i.e., those seeking breath-hold*CVR_BOLD_*values in agreement with controlled-hypercapnia*CVR_BOLD_*values) should aim for a breath-hold duration of ~10–15 s—this range is anyway expected to be more translatable to patients.

### 5.2 Breath-holding versus resting-state

Over the past two decades, researchers have claimed that*CVR_BOLD_*can be measured using low-frequency resting-state oscillations (Jahanian et al., 2014;Kannurpatti et al., 2014;Wang et al., 2016;Zang et al., 2007;Zou et al., 2008), with the rationale being that these fluctuations are temporally correlated with spontaneous$P_{ET}CO_{2}$fluctuations (Wise et al., 2004). In general, resting-state*CVR_BOLD_*can largely be grouped into signal variation methods, where signal fluctuation magnitude is considered, and regression methods, where magnitude and delay are both considered with respect to a regressor; these methods have been described at length in previous works (Golestani et al., 2016;Jahanian et al., 2014,^2017^;Lipp et al., 2015;Liu, Li, et al., 2017;Pinto et al., 2021;Poublanc et al., 2015;Zou et al., 2008;Zuo et al., 2010).

Although resting-state*CVR_BOLD_*has been shown to correlate well with controlled hypercapnia and breath-holding methods in a few studies (Jahanian et al., 2017;Liu et al., 2021), its validity as a technique for measuring CVR is unclear. In particular, the measured fluctuation magnitude, even when filtered to maximally correlate with$P_{ET}CO_{2}$fluctuations (Liu, Li, et al., 2017), is likely confounded by other processes which do not reflect CVR—most notably, neuronal activity-induced BOLD fluctuations (Smitha et al., 2017;Van den Heuvel and Hulshoff Pol, 2010).

#### 5.2.1 Experimental data

We conducted a voxel-wise statistical comparison between breath-hold and resting-state methods (using our own unpublished 3 T and 7 T data) to determine whether resting-state is systematically different from breath-hold*CVR_BOLD_*(denoted in the experimental data asCVR*), and if so, where in the brain these differences are most pronounced. The quantification ofCVR*will now be briefly described (seeSection 1of theSupplementary Materialsfor details).

Using a recently developed method to extract an arterial vasodilatory time course in response to hypercapnia (Schulman et al., 2024), we implemented a novel input regressor for both breath-hold and resting-state paradigms. The resting-state data were bandpass filtered from 0.01 to 0.045 Hz in order to isolate the frequency range of predominate resting$P_{ET}CO_{2}$fluctuations (Wise et al., 2004). For both resting-state and breath-hold paradigms, the relaxation rate time courses were linearly interpolated to a temporal resolution of 0.5 s. Time courses within the arterial regressor mask were multiplied by -1 and averaged voxel-wise to generate the arterial input regressor function (AIF). This vertical flipping of the AIF is necessary because the AIF measured in response to hypercapnia is opposite in sign relative to the tissue time courses (seeSchulman et al., 2024). TheAIFwas fit to each tissue voxel by first shifting theAIFforward in 0.5 s steps, up to a maximum of 7 s, and the correlation between theAIFand tissue time courses was recorded for each shift. The temporal shift resulting in the highest correlation was recorded as the voxel’s delay (*d*). TheAIFwas then fit to the time course in each voxel (Tissue(t)), using the voxel’s delay as an input parameter, assuming the following model:

$$Tissue\left(t\right)=CVR_{BOLD,R}^{*}\;\cdot \;AIF\left(t-d\right)+s+\varepsilon \left(t\right)$$ (11)

Here,$CVR_{BOLD,R}^{*}$is the scaling factor that minimizes least squared error between the tissue andAIFrelaxation rate time courses. In terms of the resulting maps,$CVR_{BOLD,R}^{*}$is effectively a proxy to a regression-based estimation of$CVR_{BOLD,R}$(the absolute value of$CVR_{BOLD,R}^{*}$will be different from$CVR_{BOLD,R}$as values are not normalized to$\Delta P_{ET}CO_{2}$, but comparing absolute values is not the focus of our analysis). Note that$CVR_{BOLD,R}^{*}$is not a proxy to*steady-state*$CVR_{BOLD,R}$, which requires the additional modeling of a hemodynamic response function (HRF) (Poublanc et al., 2015). Finally,$CVR_{BOLD,R}^{*}$was normalized to the average GM$CVR_{BOLD,R}^{*}$to obtain the relative CVR maps ($CVR^{*}$).srepresents the vertical shift needed to account for baseline MRI signal differences andε(t)represents the residual error.

**Fig. 6.**
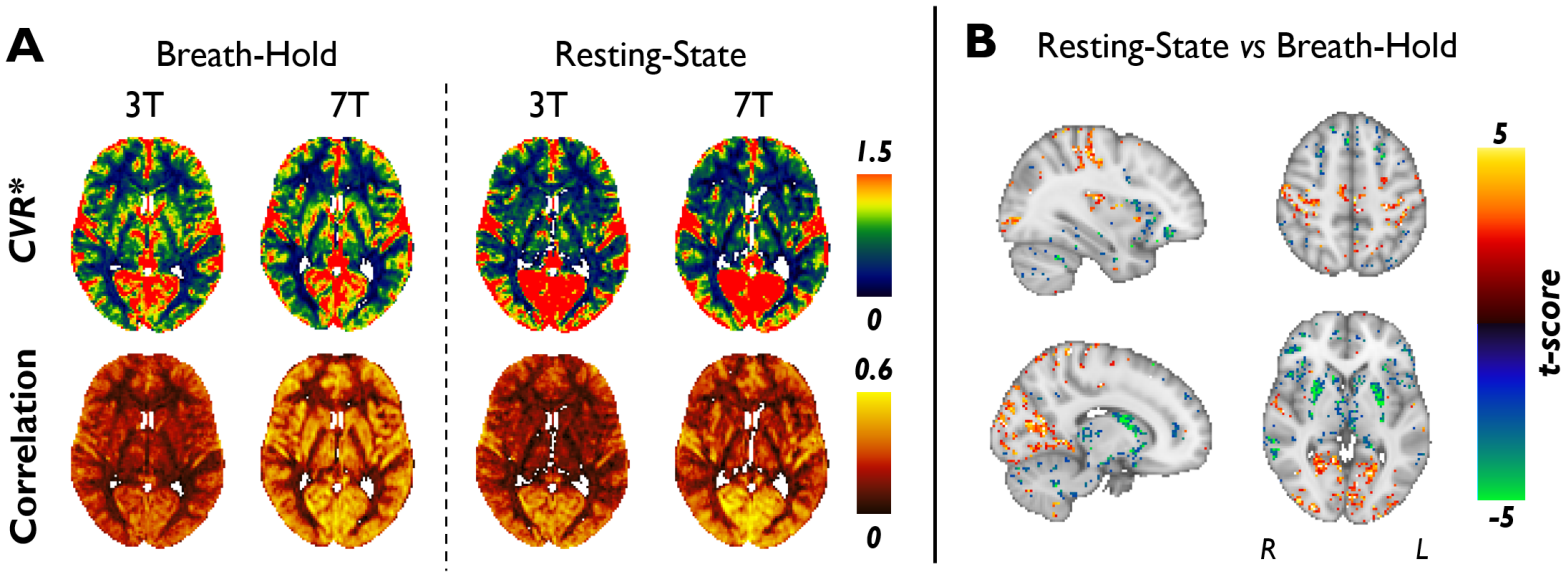
Differences between resting-state and breath-hold${\text{CVR}}^{\text{*}}$. (A)${\text{CVR}}^{\text{*}}$and input regressor correlation maps in MNI152 2 mm anatomical space for subject-averaged breath-hold and resting-state data at 3 T (n = 9) and 7 T (n = 9). (B) Statistical comparison (paired t-test, α = 0.05) between breath-hold and resting-state${\text{CVR}}^{\text{*}}$data at 7 T (seeSupplementary Fig. 3for 3 T results). Only significant t-scores are displayed; RS > BH corresponds with t > 0.

InFigure 6, the subject-averaged$CVR^{*}$maps (which are normalized to average GM) show agreement between field strengths in most regions (i.e., patterns of$CVR^{*}$values across brain regions are the same at 3 T and 7 T). Note that the same subjects were measured at both field strengths. Correlation values between the AIF regressor and voxel-wise data increase significantly from 3 T to 7 T, particularly for the breath-hold paradigm (breath-hold:*p*< 0.0005; resting-state:*p*< 0.005). InSupplementary Figure 4, the delay maps show regional agreement between breath-hold and resting-state maps. At both 3 T and 7 T, there are substantial and significant (*p<*0.05) voxel-wise differences between breath-hold and resting-state$CVR^{*}$maps (Fig. 6B). In comparison with breath-holding, resting-state yields higher$CVR^{*}$in the visual cortex, pre-central gyrus, and post-central gyrus, but lower$CVR^{*}$in the subcortical GM (e.g., putamen).

Region-specific resting-state neuronal fluctuations in the visual, motor, sensory, and default mode networks, to name a few, which are active during the resting-state (Gohel & Biswal, 2015) and similar in frequency (Buzsáki & Draguhn, 2004;Penttonen & Buzsáki, 2003;Smitha et al., 2017;Van den Heuvel and Hulshoff Pol, 2010) to CO_2_-induced relaxation fluctuations (Wise et al., 2004), are the most likely explanation for these differences between breath-hold and resting-state$CVR^{*}$maps. While these fluctuations are generally averaged out during the analysis of the breath-hold data and are smaller in amplitude than the breath-hold-induced signal changes, they are generally maintained during bandpass filtering (0.01–0.045 Hz), making it difficult to remove these contributions from resting-state$CVR^{*}$. To make matters more complex, resting-state neuronal fluctuations are highly variable between individuals and depend on their cognitive state during the scan (Gonzalez-Castillo et al., 2021;Han et al., 2023). As observed inSupplementary Figure 5, our resting-state$CVR^{*}$maps differ substantially between participants, each incorporating aspects of various resting-state networks (particularly the visual network, which is potentially due to the variability of subjects with eyes open vs. closed). The observed inter-subject variability relative to the breath-hold technique limits the generalizability of resting-state*CVR_BOLD_*. Although voxel-wise resting-state*CVR_BOLD_*data have not been widely investigated, it can be observed in Figure 4 from Liu et al. that many subjects displayed regional differences in resting-state versus CO_2_-based*CVR_BOLD_*maps (i.e., regional correlations below 0.7), with a similar observation of increased resting-state*CVR_BOLD_*in the visual cortex relative to traditional*CVR_BOLD_*(Liu et al., 2021). Although not calculated here, we also expect differences between breath-hold and resting-state$CVR^{*}$absolutevalues due to factors such as arterial compliance (Prokopiou et al., 2019).

As a final note, breath-holding-based*CVR_BOLD_*may also be confounded by activations in the brain’s respiratory centers and motor regions that are synchronized with the breath holds. In fact, a recent study found that significant BOLD activations were observed in the pontine respiratory group and raphe nuclei during breath-holding (Ciumas et al., 2023). Thus, although to a lesser extent, breath-holding-based*CVR_BOLD_*is similarly confounded by neuronal activity in certain brain regions.

#### 5.2.2 Simulations

To better understand the influence of concomitant neuronal activity on*CVR_BOLD_*quantification, we performed a very simple simulation (independent of the simulation framework inSection 2.2). Here, a tissue time course was simulated as the summation of an input CO_2_time course ($CO_{2}\left(t\right)$, simple sinusoid with a frequency of 0.033 HzWise et al., 2004), and a time course of neuronal activity (neu(t),varied in magnitude (*m*), frequency (*f*), and phase shift (*p*) relative to$CO_{2}\left(t\right)$):

$$Tissue\left(t\right)=CO_{2}\left(t\right)+neu\left(t\right)$$ (12.1)

$$Tissue\left(t\right)=sin\left(\frac{\pi }{15}\;\cdot \;t\right)+m\;\cdot \;sin\left(f\left(t-p\right)\right)$$ (12.2)

**Fig. 7.**
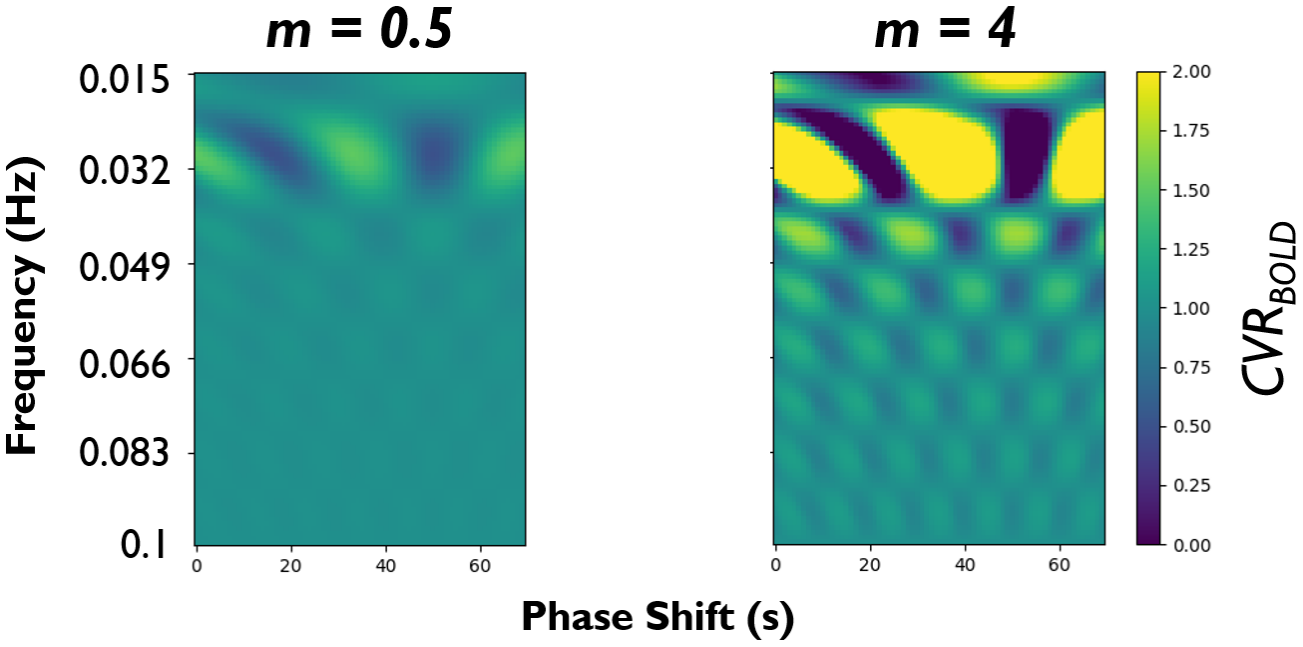
Simulated effect of resting-state neuronal activity on*CVR_BOLD_*quantification. An input CO_2_-based time course and tissue time course (summation of the input time course and a time course of neuronal activity (varied in frequency (y-axis), phase (x-axis), and magnitude (m) relative to the input time course)) were simulated.*CVR_BOLD_*was calculated as described in the experimental data (i.e., input time course was parameterized with delay and*CVR_BOLD_*(effectively a magnitude scaling factor), and least squares-based optimization was then conducted between this parameterized time course and the tissue time course).

In these simulations (Fig. 7),*CVR_BOLD_*was calculated similarly to as described inEq. 11, with$CO_{2}\left(t\right)$as the input regressor. For simplicity, a*CVR_BOLD_*of 1 indicates that quantification solely reflects the contribution of the CO_2_time course.

The simulations indicate that*CVR_BOLD_*depends on the magnitude, phase, and frequency properties of resting-state neuronal fluctuations relative to the CO_2_-based fluctuations. The influence of resting-state neuronal fluctuations on*CVR_BOLD_*quantification increases with increased resting-state neuronal fluctuation magnitude (*m*). Calculated*CVR_BOLD_*can either increase (>1) or decrease (<1) depending on the phase/frequency properties of the contaminant neuronal fluctuations. In general, the neuronal fluctuations most dramatically affect*CVR_BOLD_*estimation from 0.015 to 0.075 Hz, a typical range of activity in resting-state networks (often termed the slow-4 and slow-5 bandwidths) and similar in frequency to the simulated CO_2_fluctuations (Buzsáki & Draguhn, 2004;Penttonen & Buzsáki, 2003;Wise et al., 2004). These simulations stress the value of incorporating breath-hold modulations into the resting-state scan, as has been performed in previous work, to introduce larger CO_2_changes and minimize the relative contribution (i.e.,*m*) of neuronal activity (Liu et al., 2020;Stickland et al., 2021,^2022^).

## 6 Disease Modeling and *CVR _BOLD_*

### 6.1 Vascular occlusion and collateral flow

Of all clinical cases, steno-occlusive disease is the most widely studied using*CVR_BOLD_*(Sleight et al., 2021). Using simulations, we explored the relationship between*CVR_BOLD_*and arterial stenosis in both the presence and absence of collateral circulation (Sobczyk et al., 2020) to determine whether and to what extent*CVR_BOLD_*is representative of CVR impairment.

**Fig. 8.**
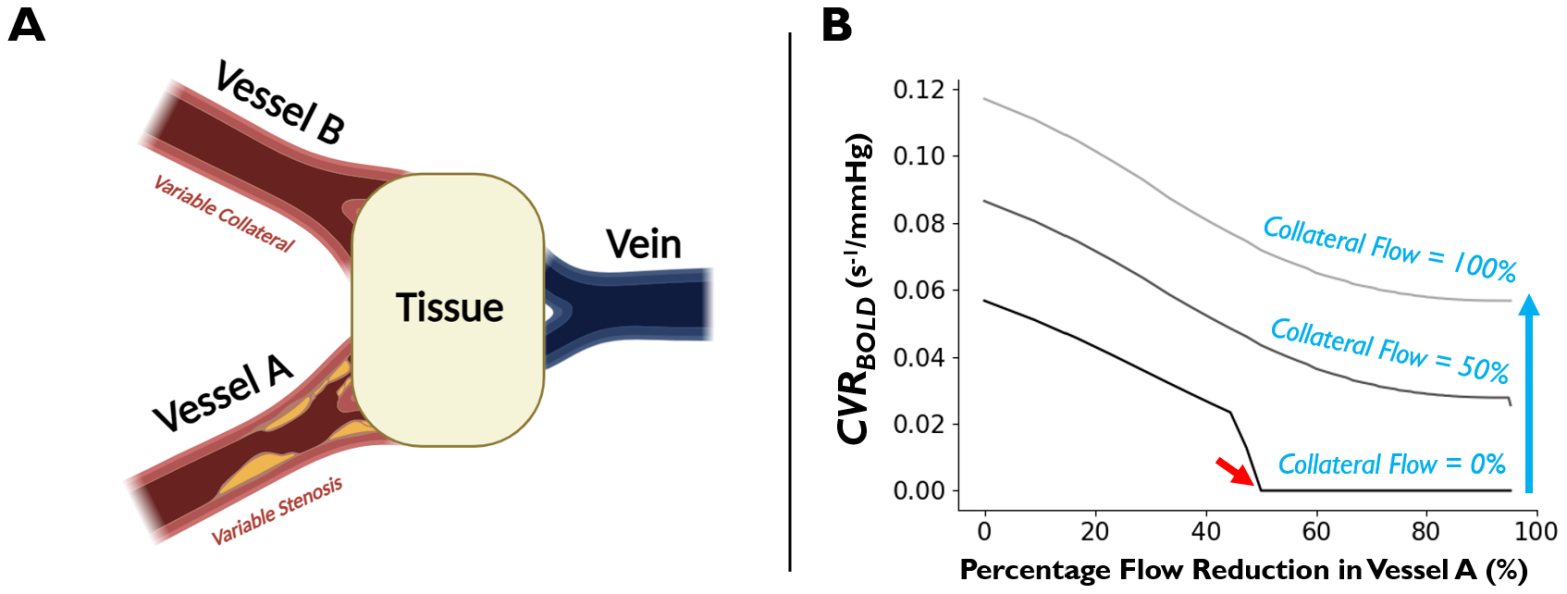
Simulated relationship between*CVR_BOLD_*estimation, vascular occlusion, and collateral flow. (A) Schematic of stenotic (Vessel A) and collateral (Vessel B) vessels supplying the imaged tissue voxel. (B)*CVR_BOLD_*(s^-1^/mmHg) for simulated tissue (downstream of Vessels A and B) that was capable of autoregulation if the cumulative upstream baseline flow (i.e., summated flow from Vessels A and B) was greater than 50% of flow in non-stenotic Vessel A. Blue arrow indicates increasing collateral flow contribution (i.e., Vessel B flow simulated from 0 to 100% of the flow supplied by a non-stenotic Vessel A). Red arrow indicates when autoregulatory capacity is reached.

In summary (see Section 2.1 of theSupplementary Materialsfor details), we simulated a tissue voxel supplied by two**independent**arterial vessels—Vessel A (with variable stenosis; x-axis) and Vessel B (with variable collateral flow; blue arrow) (Fig. 8). The CO_2_time course input to the simulated tissue is thus a summation of the dispersed CO_2_time course from Vessel A and the undispersed CO_2_time course from Vessel B. The tissue was simulated as previously described (seeSection 2.2), with one notable modification: to account for autoregulatory exhaustion, tissue vessels were only simulated to respond to the CO_2_stimulus if the cumulative upstream baseline flow (i.e., summated flow from vessels A and B) was greater than 50% of flow in non-stenotic Vessel A (Lassen, 1959).

Many studies report low*CVR_BOLD_*(Duffin et al., 2018;Hartkamp et al., 2017,^2018^;Venkatraghavan et al., 2018) and long delay/dispersion (Duffin et al., 2015;Hartkamp et al., 2012;Waddle et al., 2020) in the ipsilateral cortex of steno-occlusive disease. The simulations (Fig. 8B), prior to reaching autoregulatory capacity (i.e., before red arrow), indicate that*CVR_BOLD_*decreases due to stenosis-mediated dispersion of the input CO_2_bolus in the absence of any changes to the tissue vasculature’s physiological CVR. For example,*CVR_BOLD_*reduces by roughly 50% (i.e., from 0.057 s^-1^/mmHg to 0.028 s^-1^/mmHg) when the simulated upstream flow is reduced by 40%,**even though the tissue*****CVR_Factor_*****is unchanged**.

Dispersion effects are not only relevant in disease, but also when comparing healthy tissue with different dispersion/transit properties, such as the GM and WM. As demonstrated in DSC-MRI experiments, WM is associated with more dispersion of the input bolus (i.e., from artery to tissue) than GM (Ibaraki et al., 2007). From this, it can be expected that the observed*CVR_BOLD_*will be reduced in WM using a traditional CVR analysis, even though this dispersion-mediated reduction in bolus peak does not reflect a reduction in the tissue’s ability to vasodilate (i.e., the tissue is simply seeing a reduced CO_2_peak due to dispersion and will yield a smaller response accordingly). Given this*CVR_BOLD_*dependency, it is recommended that researchers calculate the steady-state*CVR_BOLD_*by implementing a model-based hemodynamic response function (Poublanc et al., 2015) which minimizes the dispersion confound, or calculate*CVR_BOLD_*as the integral of the*CVR_BOLD_*time course—a potential model-free, computationally inexpensive alternative, similar to the*CBV_0_*calculation in DSC-MRI (Bjørnerud & Emblem, 2010;Meier & Zierler, 1954;Ostergaard et al., 1996).

Once the tissue arterioles reach autoregulatory capacity (Fig. 8B, red arrow),*CVR_BOLD_*is 0. In fact, although not shown in this simulation, the co-occurrence of steal physiology could even yield a negative*CVR_BOLD_*measurement when the tissue arterioles have reached autoregulatory capacity (Duffin et al., 2017;Fisher & Mikulis, 2021;Sobczyk et al., 2014).

The degree of stenosis-mediated*CVR_BOLD_*reduction is heavily modulated by the degree of collateral flow (i.e., in a scenario where collateral flow replaces the entirety of flow from Vessel A,*CVR_BOLD_*would be in agreement with*CVR_BOLD_*in the absence of both occlusion and collateral flow). This agrees with the literature, where collateral flow has been posited as an explanation for the counterintuitive positive*CVR_BOLD_*observed in vascular territories affected by steno-occlusion (Sobczyk et al., 2020). Of note, increased collateral flow helps prevent the simulated tissue from reaching autoregulatory capacity (seeFig. 8B, where vasodilatory exhaustion does not occur with collateral flow greater than 50% of flow relative to non-stenotic Vessel A).

In sum, the simulations indicate that*CVR_BOLD_*may reflect physiological CVR impairment when tissue vessels have reached autoregulatory capacity, but not in the case where there is a remaining vasodilatory reserve due to stenosis-mediated bolus dispersion; this limitation may be partially attenuated by using more advanced*CVR_BOLD_*analysis strategies that model the effects of dispersion (Bjørnerud & Emblem, 2010;Meier & Zierler, 1954;Ostergaard et al., 1996;Poublanc et al., 2015).

### 6.2 Steal physiology

**Fig. 9.**
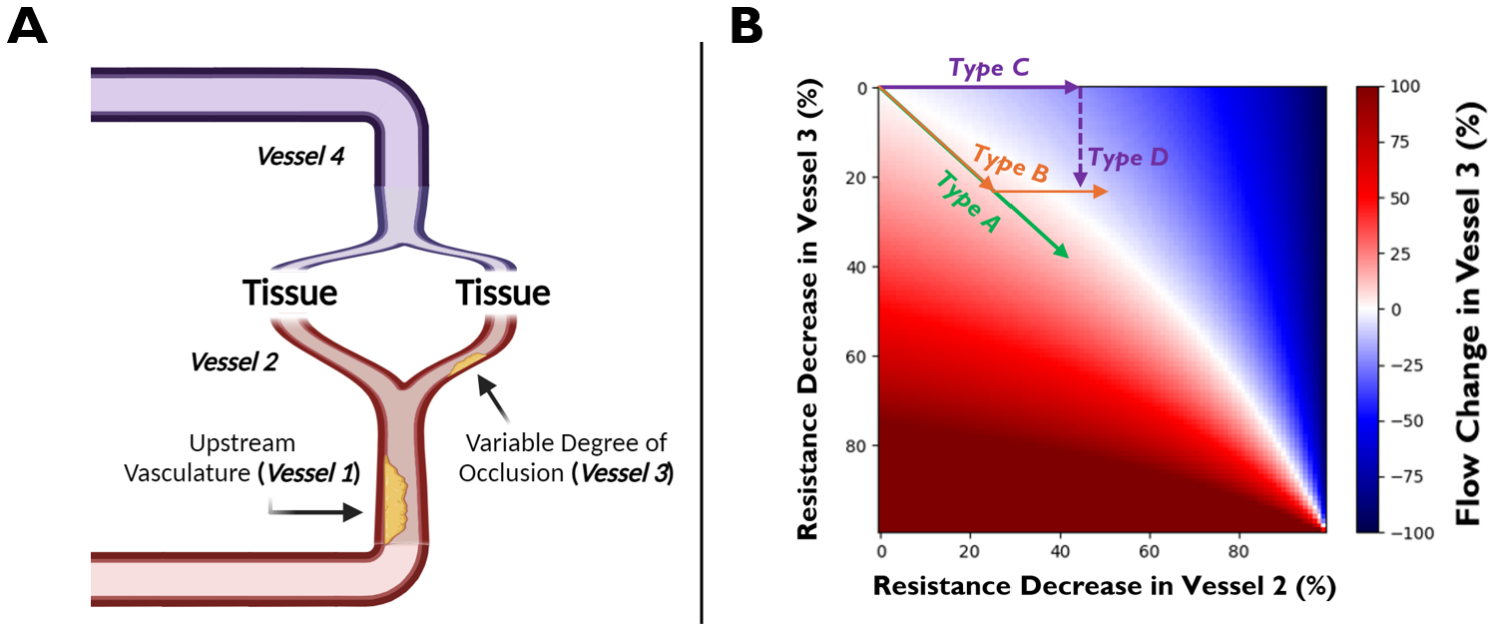
Steal physiology: Simulated relationship between vascular reactivity and flow change. (A) Schematic of steal physiology. Upstream vasculature unable to dilate due to stenosis. Vessel 3 vasodilatory capacity depends on the level of downstream stenosis. (B) Percent flow change in Vessel 3 (in the presence of a vasodilative agent) as a function of vascular reactivity in Vessel 3 (y-axis) and vascular reactivity in Vessel 2 (x-axis). Four different types of CVR responses are indicated here (see text).

Steal physiology describes the phenomenon in which vasodilatory capacity is exhausted in one vessel, such that parallel, unaffected vessels “steal” blood flow away from the exhausted vessel when presented with a vasodilatory stimulus (Brawley, 1968;Symon, 1968).*CVR_BOLD_*has often been used to detect steal physiology in the brain, where negative*CVR_BOLD_*values are often assumed to indicate regions where the blood vessels see a reduction in flow in the presence of a vasodilatory stimulus (Fisher & Mikulis, 2021;Sobczyk et al., 2014). It is not entirely clear to what extent*CVR_BOLD_*-measured steal physiology reflects ground-truth CVR impairments. To address this, we simulated steal physiology as a circuit with parallel flow and resistance (Fig. 9AandSupplementary Fig. 7). Note that these simulations are completely independent from those described inSection 2.2.

In summary (see Section 2.2 of the Supplementary Materials for details), this simulation was set up as a parallel flow circuit (Fig. 9AandSupplementary Fig. 7). Baseline resistances in Vessels 2 and 3 were set to 1; baseline resistances in Vessels 1 and 4, which represents a summation of vasculature prior to and after the parallel circuit, respectively, were set to 1/3. As “voltage” was not found to affect the quantitative values in this simulation, it was set to 1. Using Kirchoff’s laws, the resulting flow through Vessel 3 (and thus, the supplied tissue vasculature) was calculated iteratively by reducing resistance (from 100 to 0%, in 1% steps) in Vessels 2 and 3 (x- and y-axis, respectively, inFig, 9B). We assume that Vessel 4 (i.e., venous vasculature) sees a negligible resistance change relative to the other vessels (Grubb et al., 1974;Ito et al., 2003) and Vessel 1, due to simulated occlusion, also sees a negligible change—for the scenario where Vessel 1 is capable of vasodilation, seeSupplementary Figure 8.

InFigure 9B, note that positive percent flow change corresponds to positive$CVR_{CBF}$in tissue supplied by Vessel 3, whereas negative percent flow change corresponds to negative$CVR_{CBF}$in tissue supplied by Vessel 3. For simplicity here, the “sign” of$CVR_{CBF}$can be assumed to be the same sign for*CVR_BOLD_*; therefore, flow change can be thought of as a proxy to*CVR_BOLD_*.

Reducing resistance in Vessel 2 (via CO_2_-mediated dilation) while maintaining constant resistance in Vessel 3 (i.e., due to exhausted vasodilatory reserve) yields a negative flow change (i.e., steal physiology) in Vessel 3, and thus, negative*CVR_BOLD_*; however, this is not necessarily the case in a scenario where upstream vasculature is capable of vasodilation (Supplementary Fig. 8A). In particular, flow may be relatively unchanged in Vessel 3, even up to a ~40% resistance drop in Vessel 2 (Supplementary Fig. 8B), which is a typical resistance change observed during hypercapnia (Aslan et al., 2010;Blockley et al., 2013;Bulte et al., 2012;De Vis et al., 2015;Gauthier et al., 2013;Sasse et al., 1996;Vestergaard & Larsson, 2019). Thus, any observed negative*CVR_BOLD_*in this scenario would not be a result of steal physiology but may instead result from a low contrast-to-noise ratio, vasodilation-mediated negative signal change (Thomas et al., 2013), or errors in analysis (such as not performing lag optimization;Stickland et al., 2021). However, when the upstream vasculature is entirely incapable of vasodilation (Fig. 9B), steal physiology is more likely to be observed, as any resistance decrease in Vessel 2 will yield a negative flow change in Vessel 3, assuming Vessel 3 is incapable of vasodilation.

Interestingly, a steal-mediated decrease in flow may accompany an increase in blood volume for a vessel with some remaining vasodilatory reserve. For example, a 10% resistance decrease in Vessel 3 (i.e., percent blood volume increase of ~2.5%) yields a negative flow change (i.e., negative*CVR_BOLD_*) when Vessel 2 resistance decreases by 25% or more (Fig. 9B). Therefore, when studying steal physiology, it is important to identify which representation of physiological CVR (i.e.,Eqs. 1.1–1.3) is of greater interest, as agreement between the measures is not guaranteed (e.g.,*CVR_VASO_*could yield a positive result when*CVR_ASL_*yields a negative result).

The simulations provide an explanation for all four types of CVR responses which have been documented in previous studies (Duffin et al., 2017;Sobczyk et al., 2014): type A response (i.e., positive flow change with increasing CO_2_), type B response (i.e., positive flow change with increasing CO_2_, followed by negative flow change with further CO_2_increases), type C response (i.e., negative flow change with increasing CO_2_), and type D response (i.e., negative flow change with increasing CO_2_, followed by positive flow change with further CO_2_increases) (Fig. 9B).

The simulations demonstrate that*CVR_BOLD_*in steal physiology depends on the vasodilatory capacity of multiple vessels, influenced by their respective degrees of stenosis. Importantly, the simulations also demonstrate that*CVR_BOLD_*may not always accurately reflect the ground-truth CVR properties of the imaged vessels.

## 7 Limitations

In this paper, we used a simulation framework (modified from prior fMRI/DSC studies;Kjolby et al., 2006,^2009^;Schulman et al., 2022,^2023^;Uludağ et al., 2009) to understand how*CVR_BOLD_*is influenced by various physiological and physical parameters. Although the model is quite comprehensive, there are a few notable limitations that we would like to address. First, in our simulations, we assume that CMRO_2_remains constant during mild/moderate hypercapnic challenges, in agreement with findings from previous work (Blockley et al., 2013;Chen & Pike, 2010;Jain et al., 2011;Vestergaard & Larsson, 2019;Zappe et al., 2008). These studies indicate that CMRO_2_does not significantly change during hypercapnia. Fortunately, our modeling framework of*CVR_BOLD_*can easily accommodate CMRO_2_changes if needed for certain subjects or experimental conditions (in particular,Eq. 6.1). Second, we simulated tissue composed of arteriole, capillary, and venule. However, cerebral tissue may also contain contributions from larger arteries and veins. Future studies focused on understanding the influence of larger vessels on*CVR_BOLD_*can still implement our model by incorporatingEqs. 10–11fromUludağ et al., 2009for vessel diameters ≥ 200 μm. Third, we assume a ~40%$\Delta CBF_{p}$in response to a$\Delta P_{ET}CO_{2}$of 9.25 mmHg, based on previous relationships determined experimentally (Grüne et al., 2015;Poulin et al., 1996;Ramsay et al., 1993;Sato et al., 2012;Tancredi & Hoge, 2013). From study-to-study, the input$\Delta P_{ET}CO_{2}$stimulus may differ substantially from 9.25 mmHg (e.g., in resting-state vs. breath-hold vs. controlled hypercapnia). Therefore, future studies looking to simulate a smaller/larger$\Delta P_{ET}CO_{2}$with our model will need to modify$\Delta CBF_{p}$based on the relationships described in the literature (Grüne et al., 2015;Poulin et al., 1996;Ramsay et al., 1993;Sato et al., 2012;Tancredi & Hoge, 2013). Implementing other$\Delta CBF_{p}$values will change quantitative values but not alter the qualitative results/findings. Finally, it should be noted that although our simulation framework works as a forward model, using it as an inverse model (i.e., as a replacement forEq. 3for the calculation of*CVR_BOLD_*) would likely not be possible unless one were to estimate multiple physiological measures (i.e.,*CBV_0_*,*Hct*, and*Y_0_*) independently from the*CVR_BOLD_*acquisition.

In addition to our simulated work, we collected empirical breath-hold and resting-state data to understand whether*CVR_BOLD_*is concordant between these methods. There are two notable limitations with respect to the empirical portion of this work. First,$\Delta P_{ET}CO_{2}$data were not available for collection in this study—instead, we used an AIF time course as our input regressor. Scaling signal/relaxation change by$\Delta P_{ET}CO_{2}$is important for obtaining absolute*CVR_BOLD_*values. However, it should be noted that in this work, we were not interested in comparing absolute values; rather, we were interested in performing a qualitative voxel-wise comparison between resting-state and breath-hold maps (we do not expect$\Delta P_{ET}CO_{2}$scaling to confound our relative maps). Second, we did not instruct participants to keep their eyes closed or open during the resting-state scan. As has been observed in previous work (Han et al., 2023), this parameter is known to influence resting-state neuronal activity measured with fMRI, which may have contributed to some of the inter-subject variability in our study.

## 8 Summary

In this work, we sought to understand the differences between several formulations of physiological CVR (i.e., *CVR _CBV_* , *CVR _CBF_* , and *CVR _v_* ) and holistically address the accuracy of *CVR _BOLD_* . To do so, we developed a *CVR _BOLD_* simulation framework and interrogated the *CVR _BOLD_* literature to address whether and to what extent *CVR _BOLD_* accurately reflects physiological CVR, and with which parameters *CVR _BOLD_* varies the most. In summary, we have shown the following:

- The various formulations of physiological CVR (*CVR_CBV_*,*CVR_CBF_*, and*CVR_v_*) will not yield the same absolute values, and they do not yield the same relative maps in the presence of regional variations in the composition of blood vessels.
- *CVR_BOLD_*is linearly dependent on the echo time and non-linearly dependent on the magnetic field strength; quantifying*CVR_BOLD_*as*CVR_BOLD,R_*largely circumvents the dependency on echo time, while implementing an empirically validated correction factor should minimize the dependency on field strength.
- *CVR_BOLD_*is dependent on several physiological parameters, including the baseline blood volume, baseline oxygen saturation, hematocrit, and relative contribution of arterial blood; various correction factor-based approaches, which may require acquiring additional physiological data, can be utilized to minimize the influence of these parameters.
- Breath-hold-based*CVR_BOLD_*may be limited by a competing hypoxic effect; the magnitude of hypoxia increases with breath-hold duration, suggesting that short–moderate breath-hold durations (~15–20 s) will yield a decent balance between increasing the contrast-to-noise ratio and limiting the hypoxic effect.
- Resting-state-based*CVR_BOLD_*is confounded by the contribution of neuronal activity, possibly creating a discrepancy with*CVR_BOLD_*maps obtained using other approaches, such as breath-holding; this contribution can likely be reduced by introducing even basic breathing maneuvers during the scan.
- In stenotic disease,*CVR_BOLD_*is limited by stenosis-mediated dispersion, which is attenuated by the degree of collateral flow; using a*CVR_BOLD_*model which incorporates a localized hemodynamic response function can minimize this limitation.
- Negative*CVR_BOLD_*cannot always be attributed to steal physiology; likewise, positive*CVR_BOLD_*should not always be interpreted as an absence of steal physiology.

In shedding light on these quantitative nuances and providing novel solutions to circumvent some of these problems, we are optimistic that this work will help researchers and physicians more appropriately quantify and interpret CVR in both health and disease.

## Supplementary Material

Supplementary Material

## Data Availability

The dataset used for the current study is available from the corresponding authors upon reasonable request. Simulation code can be found at the following GitHub link: https://github.com/JSchul1998/CVR_Scripts

## Footnotes

* Note that CVR is reported as a percentage (Donahue et al., 2009;Lu et al., 2003;Taneja et al., 2020;Zhao et al., 2021) due to a dependency on the underlying baseline measures. That is, if baseline*CBV*($CBV_{0}$) in vessel A is twice as high as$CBV_{0}$in vessel B, there is double the vasoactive moiety in vessel A and double the blood volume change in absolute physiological units; thus, to ensure that the calculated CVR is the same in both vessels, values must be normalized to their respective baseline values.

† A proportionality coefficient may be required if other units are chosen (e.g., when using mL for*CBV*, m/s for*v*, and mL/100 g/min for*CBF*).

## References

[b1] Afzali-Hashemi , L. , Baas , K. P. A. , Schrantee , A. , Coolen , B. F. , van Osch , M. J. P. , Spann , S. M. , Nur , E. , Wood , J. C. , Biemond , B. J. , & Nederveen , A. J. ( 2021 ). Impairment of cerebrovascular hemodynamics in patients with severe and milder forms of sickle cell disease . Frontiers in Physiology , 12 , 645205 . 10.3389/fphys.2021.645205 33959037 PMC8093944

[b2] Aslan , S. , Xu , F. , Wang , P. L. , Uh , J. , Yezhuvath , U. S. , van Osch , M. , & Lu , H. ( 2010 ). Estimation of labeling efficiency in pseudocontinuous arterial spin labeling . Magnetic Resonance in Medicine , 63 ( 3 ), 765 – 771 . 10.1002/mrm.22245 20187183 PMC2922009

[b3] Attwell , D. , Buchan , A. M. , Charpak , S. , Lauritzen , M. , MacVicar , B. A. , & Newman , E. A. ( 2010 ). Glial and neuronal control of brain blood flow . Nature , 468 ( 7321 ), 232 – 243 . 10.1038/nature09613 21068832 PMC3206737

[b4] Atwi , S. , Shao , H. , Crane , D. E. , da Costa , L. , Aviv , R. I. , Mikulis , D. J. , Black , S. E. , & MacIntosh , B. J. ( 2019 ). BOLD-based cerebrovascular reactivity vascular transfer function isolates amplitude and timing responses to better characterize cerebral small vessel disease . NMR in Biomedicine , 32 ( 3 ), e4064 . 10.1002/nbm.4064 30693582

[b5] Bandettini , P. A. , Wong , E. C. , Hinks , R. S. , Tikofsky , R. S. , & Hyde , J. S. ( 1992 ). Time course EPI of human brain function during task activation . Magnetic Resonance in Medicine , 25 ( 2 ), 390 – 397 . 10.1002/mrm.1910250220 1614324

[b6] Bhogal , A. A. , Philippens , M. E. P. , Siero , J. C. W. , Fisher , J. A. , Petersen , E. T. , Luijten , P. R. , & Hoogduin , H. ( 2015 ). Examining the regional and cerebral depth-dependent BOLD cerebrovascular reactivity response at 7T . NeuroImage , 114 , 239 – 248 . 10.1016/j.neuroimage.2015.04.014 25876215

[b7] Bhogal , A. A. , Siero , J. C. W. , Fisher , J. A. , Froeling , M. , Luijten , P. , Philippens , M. , & Hoogduin , H. ( 2014 ). Investigating the non-linearity of the BOLD cerebrovascular reactivity response to targeted hypo/hypercapnia at 7T . NeuroImage , 98 , 296 – 305 . 10.1016/j.neuroimage.2014.05.006 24830840

[b8] Billett , H. H. ( 1990 ). Hemoglobin and hematocrit . In H. K. Walker , W. D. Hall , & J. W. Hurst (Eds.), Clinical methods: The history, physical, and laboratory examinations ( 3rd ed.). Butterworths . http://www.ncbi.nlm.nih.gov/books/NBK259/ 21250045

[b9] Bjørnerud , A. , & Emblem , K. E. ( 2010 ). A fully automated method for quantitative cerebral hemodynamic analysis using DSC-MRI . Journal of Cerebral Blood Flow and Metabolism: Official Journal of the International Society of Cerebral Blood Flow and Metabolism , 30 ( 5 ), 1066 – 1078 . 10.1038/jcbfm.2010.4 20087370 PMC2949177

[b200] Blockley , N. P. , Driver , I. D. , Francis , S. T. , Fisher , J. A. , & Gowland , P. A. ( 2011 ). An improved method for acquiring cerebrovascular reactivity maps . Magnetic Resonance in Medicine , 65 ( 5 ), 1278 – 1286 . 10.1002/mrm.22719 21500256

[b10] Blockley , N. P. , Griffeth , V. E. M. , Simon , A. B. , & Buxton , R. B. ( 2013 ). A review of calibrated blood oxygenation level-dependent (BOLD) methods for the measurement of task-induced changes in brain oxygen metabolism . NMR in Biomedicine , 26 ( 8 ), 987 – 1003 . 10.1002/nbm.2847 22945365 PMC3639302

[b11] Brawley , B. W. ( 1968 ). The pathophysiology of intracerebral steal following carbon dioxide inhalation, an experimental study . Scandinavian Journal of Clinical and Laboratory Investigation , 21 ( Suppl. 102 ), XIII:B . 10.3109/00365516809169045 4283868

[b12] Bright , M. G. , Bianciardi , M. , Zwart , J. A. , Murphy , K. , & Duyn , J. H. ( 2014 ). Early anti-correlated BOLD signal changes of physiologic origin . NeuroImage , 87 , 287 – 296 . 10.1016/j.neuroimage.2013.10.055 24211818 PMC4001078

[b13] Bright , M. G. , & Murphy , K. ( 2013 ). Reliable quantification of BOLD fMRI cerebrovascular reactivity despite poor breath-hold performance . NeuroImage , 83 , 559 – 568 . 10.1016/j.neuroimage.2013.07.007 23845426 PMC3899001

[b14] Bulte , D. P. , Kelly , M. , Germuska , M. , Xie , J. , Chappell , M. A. , Okell , T. W. , Bright , M. G. , & Jezzard , P. ( 2012 ). Quantitative measurement of cerebral physiology using respiratory-calibrated MRI . NeuroImage , 60 ( 1 ), 582 – 591 . 10.1016/j.neuroimage.2011.12.017 22209811 PMC7100043

[b15] Burley , C. V. , Francis , S. T. , Thomas , K. N. , Whittaker , A. C. , Lucas , S. J. E. , & Mullinger , K. J. ( 2021 ). Contrasting measures of cerebrovascular reactivity between MRI and Doppler: A cross-sectional study of younger and older healthy individuals . Frontiers in Physiology , 12 , 656746 . 10.3389/fphys.2021.656746 33912073 PMC8072486

[b16] Buxton , R. B. , Uludağ , K. , Dubowitz , D. J. , & Liu , T. T. ( 2004 ). Modeling the hemodynamic response to brain activation . NeuroImage , 23 , S220 – S233 . 10.1016/j.neuroimage.2004.07.013 15501093

[b17] Buzsáki , G. , & Draguhn , A. ( 2004 ). Neuronal oscillations in cortical networks . Science (New York, N.Y.) , 304 ( 5679 ), 1926 – 1929 . 10.1126/science.1099745 15218136

[b18] Chan , E. D. , Chan , M. M. , & Chan , M. M. ( 2013 ). Pulse oximetry: Understanding its basic principles facilitates appreciation of its limitations . Respiratory Medicine , 107 ( 6 ), 789 – 799 . 10.1016/j.rmed.2013.02.004 23490227

[b19] Chen , J. J. , & Pike , G. B. ( 2010 ). MRI measurement of the BOLD-specific flow-volume relationship during hypercapnia and hypocapnia in humans . NeuroImage , 53 ( 2 ), 383 – 391 . 10.1016/j.neuroimage.2010.07.003 20624474

[b20] Ciumas , C. , Bolay , M. , Bouet , R. , Rheims , S. , Ibarrola , D. , Hampson , J. P. , Lhatoo , S. D. , & Ryvlin , P. ( 2023 ). Subject-specific activation of central respiratory centers during breath-holding functional magnetic resonance imaging . Respiration , 102 ( 4 ), 274 – 286 . 10.1159/000529388 36750046

[b21] Cohen , A. D. , & Wang , Y. ( 2019 ). Improving the assessment of breath-holding induced cerebral vascular reactivity using a multiband multi-echo ASL/BOLD sequence . Scientific Reports , 9 ( 1 ), 5079 . 10.1038/s41598-019-41199-w 30911056 PMC6434035

[b22] Davis , T. L. , Kwong , K. K. , Weisskoff , R. M. , & Rosen , B. R. ( 1998 ). Calibrated functional MRI: Mapping the dynamics of oxidative metabolism . Proceedings of the National Academy of Sciences of the United States of America , 95 ( 4 ), 1834 – 1839 . 10.1073/pnas.95.4.1834 9465103 PMC19199

[b23] De Vis , J. B. , Hendrikse , J. , Bhogal , A. , Adams , A. , Kappelle , L. J. , & Petersen , E. T. ( 2015 ). Age-related changes in brain hemodynamics; A calibrated MRI study . Human Brain Mapping , 36 ( 10 ), 3973 – 3987 . 10.1002/hbm.22891 26177724 PMC6869092

[b24] Dengel , D. R. , Evanoff , N. G. , Marlatt , K. L. , Geijer , J. R. , Mueller , B. A. , & Lim , K. O. ( 2017 ). Reproducibility of blood oxygen level-dependent signal changes with end-tidal carbon dioxide alterations . Clinical Physiology and Functional Imaging , 37 ( 6 ), 794 – 798 . 10.1111/cpf.12358 26934185 PMC5857354

[b25] Dlamini , N. , Shah-Basak , P. , Leung , J. , Kirkham , F. , Shroff , M. , Kassner , A. , Robertson , A. , Dirks , P. , Westmacott , R. , deVeber , G. , & Logan , W. ( 2018 ). Breath-hold blood oxygen level–dependent MRI: A tool for the assessment of cerebrovascular reserve in children with moyamoya disease . AJNR: American Journal of Neuroradiology , 39 ( 9 ), 1717 – 1723 . 10.3174/ajnr.A5739 30139753 PMC7655282

[b26] Donahue , M. J. , Dethrage , L. M. , Faraco , C. C. , Jordan , L. C. , Clemmons , P. , Singer , R. , Mocco , J. , Shyr , Y. , Desai , A. , O’Duffy , A. , Riebau , D. , Hermann , L. , Connors , J. , Kirshner , H. , & Strother , M. K. ( 2014 ). Routine clinical evaluation of cerebrovascular reserve capacity using carbogen in patients with intracranial stenosis . Stroke , 45 ( 8 ), 2335 – 2341 . 10.1161/STROKEAHA.114.005975 24938845 PMC4118584

[b27] Donahue , M. J. , Stevens , R. D. , de Boorder , M. , Pekar , J. J. , Hendrikse , J. , & van Zijl , P. C . ( 2009 ). Hemodynamic changes after visual stimulation and breath holding provide evidence for an uncoupling of cerebral blood flow and volume from oxygen metabolism . Journal of Cerebral Blood Flow & Metabolism , 29 ( 1 ), 176 – 185 . 10.1038/jcbfm.2008.109 18797471 PMC2865199

[b28] Driver , I. , Blockley , N. , Fisher , J. , Francis , S. , & Gowland , P. ( 2010 ). The change in cerebrovascular reactivity between 3 T and 7 T measured using graded hypercapnia . NeuroImage , 51 ( 1 ), 274 – 279 . 10.1016/j.neuroimage.2009.12.113 20056163

[b29] Duffin , J. , Sobczyk , O. , Crawley , A. P. , Poublanc , J. , Mikulis , D. J. , & Fisher , J. A. ( 2015 ). The dynamics of cerebrovascular reactivity shown with transfer function analysis . NeuroImage , 114 , 207 – 216 . 10.1016/j.neuroimage.2015.04.029 25891374

[b30] Duffin , J. , Sobczyk , O. , Crawley , A. , Poublanc , J. , Venkatraghavan , L. , Sam , K. , Mutch , A. , Mikulis , D. , & Fisher , J. ( 2017 ). The role of vascular resistance in BOLD responses to progressive hypercapnia . Human Brain Mapping , 38 ( 11 ), 5590 – 5602 . 10.1002/hbm.23751 28782872 PMC6866756

[b31] Duffin , J. , Sobczyk , O. , McKetton , L. , Crawley , A. , Poublanc , J. , Venkatraghavan , L. , Sam , K. , Mutch , W. A. , Mikulis , D. , & Fisher , J. A. ( 2018 ). Cerebrovascular resistance: The basis of cerebrovascular reactivity . Frontiers in Neuroscience , 12 , 409 . 10.3389/fnins.2018.00409 29973862 PMC6020782

[b32] Echevarria , C. , Steer , J. , Wason , J. , & Bourke , S. ( 2021 ). Oxygen therapy and inpatient mortality in COPD exacerbation . Emergency Medicine Journal , 38 ( 3 ), 170 – 177 . 10.1136/emermed-2019-209257 33243839

[b33] Fierstra , J. , Sobczyk , O. , Battisti-Charbonney , A. , Mandell , D. M. , Poublanc , J. , Crawley , A. P. , Mikulis , D. J. , Duffin , J. , & Fisher , J. A. ( 2013 ). Measuring cerebrovascular reactivity: What stimulus to use? The Journal of Physiology , 591 ( Pt 23 ), 5809 – 5821 . 10.1113/jphysiol.2013.259150 24081155 PMC3872753

[b34] Fierstra , J. , van Niftrik , C. , Piccirelli , M. , Bozinov , O. , Pangalu , A. , Krayenbühl , N. , Valavanis , A. , Weller , M. , & Regli , L. ( 2018 ). Diffuse gliomas exhibit whole brain impaired cerebrovascular reactivity . Magnetic Resonance Imaging , 45 , 78 – 83 . 10.1016/j.mri.2017.09.017 28986176

[b35] Fisher , J. A. , & Mikulis , D. J. ( 2021 ). Cerebrovascular reactivity: Purpose, optimizing methods, and limitations to interpretation—A personal 20-year odyssey of (re)searching . Frontiers in Physiology , 12 , 629651 . https://www.frontiersin.org/articles/10.3389/fphys.2021.629651 33868001 10.3389/fphys.2021.629651 PMC8047146

[b36] Fisher , J. A. , Venkatraghavan , L. , & Mikulis , D. J. ( 2018 ). Magnetic resonance imaging–based cerebrovascular reactivity and hemodynamic reserve . Stroke , 49 ( 8 ), 2011 – 2018 . 10.1161/STROKEAHA.118.021012 29986929

[b37] Gauthier , C. J. , Madjar , C. , Desjardins-Crépeau , L. , Bellec , P. , Bherer , L. , & Hoge , R. D. ( 2013 ). Age dependence of hemodynamic response characteristics in human functional magnetic resonance imaging . Neurobiology of Aging , 34 ( 5 ), 1469 – 1485 . 10.1016/j.neurobiolaging.2012.11.002 23218565

[b38] Gohel , S. R. , & Biswal , B. B. ( 2015 ). Functional integration between brain regions at rest occurs in multiple-frequency bands . Brain Connectivity , 5 ( 1 ), 23 – 34 . 10.1089/brain.2013.0210 24702246 PMC4313418

[b39] Golestani , A. M. , Wei , L. L. , & Chen , J. J. ( 2016 ). Quantitative mapping of cerebrovascular reactivity using resting-state BOLD fMRI: Validation in healthy adults . NeuroImage , 138 , 147 – 163 . 10.1016/j.neuroimage.2016.05.025 27177763 PMC5148619

[b40] Gonzalez-Castillo , J. , Kam , J. W. Y. , Hoy , C. W. , & Bandettini , P. A. ( 2021 ). How to interpret resting-state fMRI: Ask your participants . Journal of Neuroscience , 41 ( 6 ), 1130 – 1141 . 10.1523/JNEUROSCI.1786-20.2020 33568446 PMC7888219

[b41] Grubb , R. L. , Raichle , M. E. , Eichling , J. O. , & Ter-Pogossian , M. M. ( 1974 ). The effects of changes in PaCO2 on cerebral blood volume, blood flow, and vascular mean transit time . Stroke , 5 ( 5 ), 630 – 639 . 10.1161/01.str.5.5.630 4472361

[b42] Grüne , F. , Kazmaier , S. , Stolker , R. J. , Visser , G. H. , & Weyland , A. ( 2015 ). Carbon dioxide induced changes in cerebral blood flow and flow velocity: Role of cerebrovascular resistance and effective cerebral perfusion pressure . Journal of Cerebral Blood Flow & Metabolism , 35 ( 9 ), 1470 – 1477 . 10.1038/jcbfm.2015.63 25873428 PMC4640336

[b43] Han , J. , Keedy , S. , & de Wit , H . ( 2023 ). Stimulant-like subjective effects of alcohol are not related to resting-state connectivity in healthy men . Cerebral Cortex , 33 ( 16 ), 9478 – 9488 . 10.1093/cercor/bhad218 37339883 PMC10656944

[b44] Hartkamp , N. S. , Bokkers , R. P. H. , van Osch , M. J. P. , de Borst , G. J. , & Hendrikse , J. ( 2017 ). Cerebrovascular reactivity in the caudate nucleus, lentiform nucleus and thalamus in patients with carotid artery disease . Journal of Neuroradiology = Journal De Neuroradiologie , 44 ( 2 ), 143 – 150 . 10.1016/j.neurad.2016.07.003 27743788

[b45] Hartkamp , N. S. , Hendrikse , J. , van der Worp , H. B. , de Borst , G. J. , & Bokkers , R. P. H. ( 2012 ). Time course of vascular reactivity using repeated phase-contrast MR angiography in patients with carotid artery stenosis . Stroke , 43 ( 2 ), 553 – 556 . 10.1161/STROKEAHA.111.637314 22052518

[b46] Hartkamp , N. S. , Petersen , E. T. , Chappell , M. A. , Okell , T. W. , Uyttenboogaart , M. , Zeebregts , C. J. , & Bokkers , R. P. ( 2018 ). Relationship between haemodynamic impairment and collateral blood flow in carotid artery disease . Journal of Cerebral Blood Flow & Metabolism , 38 ( 11 ), 2021 – 2032 . 10.1177/0271678X17724027 28776469 PMC6238174

[b47] Havlicek , M. , Ivanov , D. , Poser , B. A. , & Uludag , K. ( 2017 ). Echo-time dependence of the BOLD response transients—A window into brain functional physiology . NeuroImage , 159 , 355 – 370 . 10.1016/j.neuroimage.2017.07.034 28729160

[b48] Hoge , R. D. , Atkinson , J. , Gill , B. , Crelier , G. R. , Marrett , S. , & Pike , G. B. ( 1999 ). Investigation of BOLD signal dependence on cerebral blood flow and oxygen consumption: The deoxyhemoglobin dilution model . Magnetic Resonance in Medicine , 42 ( 5 ), 849 – 863 . 10.1002/(SICI)1522-2594(199911)42:5<849::AID-MRM4>3.0.CO;2-Z 10542343

[b49] Hoogeveen , E. S. , Pelzer , N. , Ghariq , E. , van Osch , M. J. P. , Dahan , A. , Terwindt , G. M. , & Kruit , M. C. ( 2024 ). Cerebrovascular reactivity to hypercapnia in patients with migraine: A dual-echo arterial spin labeling MRI study . Headache: The Journal of Head and Face Pain , 64 ( 3 ), 276 – 284 . 10.1111/head.14680 38429974

[b50] Hou , X. , Liu , P. , Li , Y. , Jiang , D. , De Vis , J. B. , Lin , Z. , Sur , S. , Baker , Z. , Mao , D. , Ravi , H. , Rodrigue , K. , Albert , M. , Park , D. C. , & Lu , H. ( 2020 ). The association between BOLD-based cerebrovascular reactivity (CVR) and end-tidal CO2 in healthy subjects . NeuroImage , 207 , 116365 . 10.1016/j.neuroimage.2019.116365 31734432 PMC8080082

[b51] Ibaraki , M. , Ito , H. , Shimosegawa , E. , Toyoshima , H. , Ishigame , K. , & Takahashi , K. ( 2007 ). Cerebral vascular mean transit time in healthy humans: A comparative study with PET and dynamic susceptibility contrast-enhanced MRI . Journal of Cerebral Blood Flow & Metabolism , 27 , 404 – 413 . 10.1038/sj.jcbfm.9600337 16736045

[b52] Inoue , Y. , Tanaka , Y. , Hata , H. , & Hara , T. ( 2014 ). Arterial spin-labeling evaluation of cerebrovascular reactivity to acetazolamide in healthy subjects . AJNR: American Journal of Neuroradiology , 35 ( 6 ), 1111 – 1116 . 10.3174/ajnr.A3815 24371025 PMC7965130

[b54] Ito , H. , Kanno , I. , Ibaraki , M. , Hatazawa , J. , & Miura , S. ( 2003 ). Changes in human cerebral blood flow and cerebral blood volume during hypercapnia and hypocapnia measured by positron emission tomography . Journal of Cerebral Blood Flow & Metabolism , 23 ( 6 ), 665 – 670 . 10.1097/01.WCB.0000067721.64998.F5 12796714

[b55] Jahanian , H. , Christen , T. , Moseley , M. E. , Pajewski , N. M. , Wright , C. B. , Tamura , M. K. , & Zaharchuk , G. ( 2017 ). Measuring vascular reactivity with resting-state blood oxygenation level-dependent (BOLD) signal fluctuations: A potential alternative to the breath-holding challenge? Journal of Cerebral Blood Flow & Metabolism , 37 ( 7 ), 2526 – 2538 . 10.1177/0271678X16670921 27683452 PMC5531349

[b56] Jahanian , H. , Ni , W. W. , Christen , T. , Moseley , M. E. , Tamura , M. K. , & Zaharchuk , G. ( 2014 ). Spontaneous BOLD signal fluctuations in young healthy subjects and elderly patients with chronic kidney disease . PLoS One , 9 ( 3 ), e92539 . 10.1371/journal.pone.0092539 24651703 PMC3961376

[b57] Jain , V. , Langham , M. C. , Floyd , T. F. , Jain , G. , Magland , J. F. , & Wehrli , F. W. ( 2011 ). Rapid magnetic resonance measurement of global cerebral metabolic rate of oxygen consumption in humans during rest and hypercapnia . Journal of Cerebral Blood Flow and Metabolism: Official Journal of the International Society of Cerebral Blood Flow and Metabolism , 31 ( 7 ), 1504 – 1512 . 10.1038/jcbfm.2011.34 21505481 PMC3137470

[b58] Kannurpatti , S. S. , Motes , M. A. , Biswal , B. B. , & Rypma , B. ( 2014 ). Assessment of unconstrained cerebrovascular reactivity marker for large age-range fMRI studies . PLoS One , 9 ( 2 ), e88751 . 10.1371/journal.pone.0088751 24551151 PMC3923811

[b59] Kassner , A. , Winter , J. D. , Poublanc , J. , Mikulis , D. J. , & Crawley , A. P. ( 2010 ). Blood-oxygen level dependent MRI measures of cerebrovascular reactivity using a controlled respiratory challenge: Reproducibility and gender differences . Journal of Magnetic Resonance Imaging: JMRI , 31 ( 2 ), 298 – 304 . 10.1002/jmri.22044 20099341

[b60] Kety , S. S. , & Schmidt , C. F. ( 1948 ). The effects of altered arterial tensions of carbon dioxide and oxygen on cerebral blood flow and cerebral oxygen consumption of normal young men 1 . Journal of Clinical Investigation , 27 ( 4 ), 484 – 492 . 10.1172/jci101995 16695569 PMC439519

[b61] Kjølby , B. F. , Mikkelsen , I. K. , Pedersen , M. , Østergaard , L. , & Kiselev , V. G. ( 2009 ). Analysis of partial volume effects on arterial input functions using gradient echo: A simulation study . Magnetic Resonance in Medicine , 61 , 1300 – 1309 . 10.1002/mrm.21849 19365857

[b62] Kjølby , B. F. , Østergaard , L. , & Kiselev , V. G. ( 2006 ). Theoretical model of intravascular paramagnetic tracers effect on tissue relaxation . Magnetic Resonance in Medicine , 56 , 187 – 197 . 10.1002/mrm.20920 16724299

[b63] Kosinski , P. D. , Croal , P. L. , Leung , J. , Williams , S. , Odame , I. , Hare , G. M. T. , Shroff , M. , & Kassner , A. ( 2017 ). The severity of anaemia depletes cerebrovascular dilatory reserve in children with sickle cell disease: A quantitative magnetic resonance imaging study . British Journal of Haematology , 176 ( 2 ), 280 – 287 . 10.1111/bjh.14424 27905100

[b64] Krainik , A. , Hund-Georgiadis , M. , Zysset , S. , & von Cramon , D. Y . ( 2005 ). Regional impairment of cerebrovascular reactivity and BOLD signal in adults after stroke . Stroke , 36 ( 6 ), 1146 – 1152 . 10.1161/01.STR.0000166178.40973.a7 15879326

[b65] Kuwabara , Y. , Sasaki , M. , Hirakata , H. , Koga , H. , Nakagawa , M. , Chen , T. , Kaneko , K. , Masuda , K. , & Fujishima , M. ( 2002 ). Cerebral blood flow and vasodilatory capacity in anemia secondary to chronic renal failure . Kidney International , 61 ( 2 ), 564 – 569 . 10.1046/j.1523-1755.2002.00142.x 11849397

[b66] Kwong , K. K. , Belliveau , J. W. , Chesler , D. A. , Goldberg , I. E. , Weisskoff , R. M. , Poncelet , B. P. , Kennedy , D. N. , Hoppel , B. E. , Cohen , M. S. , & Turner , R. ( 1992 ). Dynamic magnetic resonance imaging of human brain activity during primary sensory stimulation . Proceedings of the National Academy of Sciences of the United States of America , 89 ( 12 ), 5675 – 5679 . 10.1073/pnas.89.12.5675 1608978 PMC49355

[b67] Lassen , N. A. ( 1959 ). Cerebral blood flow and oxygen consumption in man . Physiological Reviews , 39 ( 2 ), 183 – 238 . 10.1152/physrev.1959.39.2.183 13645234

[b68] Le , T. T. , Fischbein , N. J. , André , J. B. , Wijman , C. , Rosenberg , J. , & Zaharchuk , G. ( 2012 ). Identification of venous signal on arterial spin labeling improves diagnosis of dural arteriovenous fistulas and small arteriovenous malformations . AJNR: American Journal of Neuroradiology , 33 ( 1 ), 61 – 68 . 10.3174/ajnr.A2761 22158927 PMC7966149

[b69] Leung , J. , Duffin , J. , Fisher , J. A. , & Kassner , A. ( 2016 ). MRI-based cerebrovascular reactivity using transfer function analysis reveals temporal group differences between patients with sickle cell disease and healthy controls . NeuroImage: Clinical , 12 , 624 – 630 . 10.1016/j.nicl.2016.09.009 27722086 PMC5048082

[b70] Leung , J. , Kim , J. A. , & Kassner , A. ( 2016 ). Reproducibility of cerebrovascular reactivity measures in children using BOLD MRI . Journal of Magnetic Resonance Imaging , 43 ( 5 ), 1191 – 1195 . 10.1002/jmri.25063 26435493

[b71] Lipp , I. , Murphy , K. , Caseras , X. , & Wise , R. G. ( 2015 ). Agreement and repeatability of vascular reactivity estimates based on a breath-hold task and a resting state scan . NeuroImage , 113 , 387 – 396 . 10.1016/j.neuroimage.2015.03.004 25795342 PMC4441043

[b201] Liu , P. , De Vis , J. B. , & Lu , H. ( 2019 ). Cerebrovascular reactivity (CVR) MRI with CO2 challenge: A technical review . NeuroImage , 187 , 104 – 115 . 10.1016/j.neuroimage.2018.03.047 29574034 PMC6150860

[b72] Liu , P. , Li , Y. , Pinho , M. , Park , D. C. , Welch , B. G. , & Lu , H. ( 2017 ). Cerebrovascular reactivity mapping without gas challenges . NeuroImage , 146 , 320 – 326 . 10.1016/j.neuroimage.2016.11.054 27888058 PMC5321860

[b73] Liu , P. , Liu , G. , Pinho , M. C. , Lin , Z. , Thomas , B. P. , Rundle , M. , Park , D. C. , Huang , J. , Welch , B. G. , & Lu , H. ( 2021 ). Cerebrovascular reactivity mapping using resting-state BOLD functional MRI in healthy adults and patients with moyamoya disease . Radiology , 299 ( 2 ), 419 – 425 . 10.1148/radiol.2021203568 33687287 PMC8108558

[b74] Liu , P. , Welch , B. G. , Li , Y. , Gu , H. , King , D. , Yang , Y. , Pinho , M. , & Lu , H. ( 2017 ). Multiparametric imaging of brain hemodynamics and function using gas-inhalation MRI . NeuroImage , 146 , 715 – 723 . 10.1016/j.neuroimage.2016.09.063 27693197 PMC5322044

[b75] Liu , P. , Xu , C. , Lin , Z. , Sur , S. , Li , Y. , Yasar , S. , Rosenberg , P. , Albert , M. , & Lu , H. ( 2020 ). Cerebrovascular reactivity mapping using intermittent breath modulation . NeuroImage , 215 , 116787 . 10.1016/j.neuroimage.2020.116787 32278094 PMC7292765

[b76] Lu , H. , & Ge , Y. ( 2008 ). Quantitative evaluation of oxygenation in venous vessels using T2-Relaxation-Under-Spin-Tagging MRI . Magnetic Resonance in Medicine , 60 , 357 – 363 . 10.1002/mrm.21627 18666116 PMC2587050

[b77] Lu , H. , Golay , X. , Pekar , J. J. , & Van Zijl , P. C. M . ( 2003 ). Functional magnetic resonance imaging based on changes in vascular space occupancy . Magnetic Resonance in Medicine , 50 ( 2 ), 263 – 274 . 10.1002/mrm.10519 12876702

[b78] Lu , H. , & van Zijl , P. C. M . ( 2012 ). A review of the development of Vascular-Space-Occupancy (VASO) fMRI . NeuroImage , 62 ( 2 ), 736 – 742 . 10.1016/j.neuroimage.2012.01.013 22245650 PMC3328630

[b79] Mardimae , A. , Balaban , D. Y. , Machina , M. A. , Battisti-Charbonney , A. , Han , J. S. , Katznelson , R. , Minkovich , L. L. , Fedorko , L. , Murphy , P. M. , Wasowicz , M. , Naughton , F. , Meineri , M. , Fisher , J. A. , & Duffin , J. ( 2012 ). The interaction of carbon dioxide and hypoxia in the control of cerebral blood flow . Pflugers Archiv: European Journal of Physiology , 464 ( 4 ), 345 – 351 . 10.1007/s00424-012-1148-1 22961068

[b80] Meier , P. , & Zierler , K. L. ( 1954 ). On the theory of the indicator-dilution method for measurement of blood flow and volume . Journal of Applied Physiology , 6 ( 12 ), 731 – 744 . 10.1152/jappl.1954.6.12.731 13174454

[b81] Miller , K. B. , Howery , A. J. , Rivera-Rivera , L. A. , Johnson , S. C. , Rowley , H. A. , Wieben , O. , & Barnes , J. N. ( 2019 ). Age-related reductions in cerebrovascular reactivity using 4D flow MRI . Frontiers in Aging Neuroscience , 11 , 281 . 10.3389/fnagi.2019.00281 31680935 PMC6811507

[b82] Mintun , M. A. , Raichle , M. E. , Martin , W. R. , & Herscovitch , P. ( 1984 ). Brain oxygen utilization measured with O-15 radiotracers and positron emission tomography . Journal of Nuclear Medicine: Official Publication, Society of Nuclear Medicine , 25 ( 2 ), 177 – 187 . 10.1038/jcbfm.1987.97 6610032

[b83] Moia , S. , Stickland , R. C. , Ayyagari , A. , Termenon , M. , Caballero-Gaudes , C. , & Bright , M. G. ( 2020 ). Voxelwise optimization of hemodynamic lags to improve regional CVR estimates in breath-hold fMRI . In 2020 42nd Annual International Conference of the IEEE Engineering in Medicine & Biology Society (EMBC) (pp. 1489 – 1492 ). IEEE . 10.1109/EMBC44109.2020.9176225 33018273

[b84] Moia , S. , Termenon , M. , Uruñuela , E. , Chen , G. , Stickland , R. C. , Bright , M. G. , & Caballero-Gaudes , C. ( 2021 ). ICA-based denoising strategies in breath-hold induced cerebrovascular reactivity mapping with multi echo BOLD fMRI . NeuroImage , 233 , 117914 . 10.1016/j.neuroimage.2021.117914 33684602 PMC8351526

[b85] Murphy , K. , Harris , A. D. , & Wise , R. G. ( 2011 ). Robustly measuring vascular reactivity differences with breath-hold: Normalising stimulus-evoked and resting state BOLD fMRI data . NeuroImage , 54 ( 1 ), 369 – 379 . 10.1016/j.neuroimage.2010.07.059 20682354

[b86] Nöth , U. , Meadows , G. E. , Kotajima , F. , Deichmann , R. , Corfield , D. R. , & Turner , R. ( 2006 ). Cerebral vascular response to hypercapnia: Determination with perfusion MRI at 1.5 and 3.0 Tesla using a pulsed arterial spin labeling technique . Journal of Magnetic Resonance Imaging , 24 ( 6 ), 1229 – 1235 . 10.1002/jmri.20761 17094105

[b87] Nur , E. , Kim , Y.-S. , Truijen , J. , van Beers , E. J. , Davis , S. C. A. T. , Brandjes , D. P. , Biemond , B. J. , & van Lieshout , J. J . ( 2009 ). Cerebrovascular reserve capacity is impaired in patients with sickle cell disease . Blood , 114 ( 16 ), 3473 – 3478 . 10.1182/blood-2009-05-223859 19700663

[b88] Ogawa , S. , Lee , T. M. , Kay , A. R. , & Tank , D. W. ( 1990 ). Brain magnetic resonance imaging with contrast dependent on blood oxygenation . Proceedings of the National Academy of Sciences of the United States of America , 87 ( 24 ), 9868 – 9872 . 10.1073/pnas.87.24.9868 2124706 PMC55275

[b89] Ogawa , S. , Tank , D. W. , Menon , R. , Ellermann , J. M. , Kim , S. G. , Merkle , H. , & Ugurbil , K. ( 1992 ). Intrinsic signal changes accompanying sensory stimulation: Functional brain mapping with magnetic resonance imaging . Proceedings of the National Academy of Sciences of the United States of America , 89 ( 13 ), 5951 – 5955 . 10.1073/pnas.89.13.5951 1631079 PMC402116

[b90] Østergaard , L. ( 2005 ). Principles of cerebral perfusion imaging by bolus tracking . Journal of Magnetic Resonance Imaging: JMRI , 22 ( 6 ), 710 – 717 . 10.1002/jmri.20460 16261573

[b91] Ostergaard , L. , Weisskoff , R. M. , Chesler , D. A. , Gyldensted , C. , & Rosen , B. R. ( 1996 ). High resolution measurement of cerebral blood flow using intravascular tracer bolus passages. Part I: Mathematical approach and statistical analysis . Magnetic Resonance in Medicine , 36 ( 5 ), 715 – 725 . 10.1002/mrm.1910360510 8916022

[b92] Peng , S.-L. , Yang , H.-C. , Chen , C.-M. , & Shih , C.-T. ( 2020 ). Short- and long-term reproducibility of BOLD signal change induced by breath-holding at 1.5 and 3 T . NMR in Biomedicine , 33 ( 3 ), e4195 . 10.1002/nbm.4195 31885110

[b93] Penttonen , M. , & Buzsáki , G. ( 2003 ). Natural logarithmic relationship between brain oscillators . Thalamus & Related Systems , 2 ( 2 ), 145 – 152 . 10.1016/S1472-9288(03)00007-4

[b202] Petersen , E. T. , Zimine , I. , Ho , Y.-C. L. , & Golay , X. ( 2006 ). Non-invasive measurement of perfusion: A critical review of arterial spin labelling techniques . The British Journal of Radiology , 79 ( 944 ), 688 – 701 . 10.1259/bjr/67705974 16861326

[b94] Pillai , J. J. , & Zacá , D. ( 2011 ). Clinical utility of cerebrovascular reactivity mapping in patients with low grade gliomas . World Journal of Clinical Oncology , 2 ( 12 ), 397 – 403 . 10.5306/wjco.v2.i12.397 22171282 PMC3235658

[b95] Pinto , J. , Bright , M. G. , Bulte , D. P. , & Figueiredo , P. ( 2021 ). Cerebrovascular reactivity mapping without gas challenges: A methodological guide . Frontiers in Physiology , 11 , 608475 . https://www.frontiersin.org/articles/10.3389/fphys.2020.608475 33536935 10.3389/fphys.2020.608475 PMC7848198

[b96] Pinto , J. , Jorge , J. , Sousa , I. , Vilela , P. , & Figueiredo , P. ( 2016 ). Fourier modeling of the BOLD response to a breath-hold task: Optimization and reproducibility . NeuroImage , 135 , 223 – 231 . 10.1016/j.neuroimage.2016.02.037 26908316

[b97] Poublanc , J. , Crawley , A. P. , Sobczyk , O. , Montandon , G. , Sam , K. , Mandell , D. M. , Dufort , P. , Venkatraghavan , L. , Duffin , J. , Mikulis , D. J. , & Fisher , J. A. ( 2015 ). Measuring cerebrovascular reactivity: The dynamic response to a step hypercapnic stimulus . Journal of Cerebral Blood Flow & Metabolism , 35 ( 11 ), 1746 – 1756 . 10.1038/jcbfm.2015.114 26126862 PMC4635229

[b98] Poublanc , J. , Sobczyk , O. , Shafi , R. , Sayin , E. S. , Schulman , J. , Duffin , J. , Uludag , K. , Wood , J. C. , Vu , C. , Dharmakumar , R. , Fisher , J. A. , & Mikulis , D. J. ( 2021 ). Perfusion MRI using endogenous deoxyhemoglobin as a contrast agent: Preliminary data . Magnetic Resonance in Medicine , 86 ( 6 ), 3012 – 3021 . 10.1002/mrm.28974 34687064

[b99] Poulin , M. J. , Liang , P. J. , & Robbins , P. A. ( 1996 ). Dynamics of the cerebral blood flow response to step changes in end-tidal PCO2 and PO2 in humans . Journal of Applied Physiology (Bethesda, Md.: 1985) , 81 ( 3 ), 1084 – 1095 . 10.1152/jappl.1996.81.3.1084 8889738

[b100] Prokopiou , P. C. , Pattinson , K. T. S. , Wise , R. G. , & Mitsis , G. D. ( 2019 ). Modeling of dynamic cerebrovascular reactivity to spontaneous and externally induced CO2 fluctuations in the human brain using BOLD-fMRI . NeuroImage , 186 , 533 – 548 . 10.1016/j.neuroimage.2018.10.084 30423427

[b101] Ramsay , S. C. , Murphy , K. , Shea , S. A. , Friston , K. J. , Lammertsma , A. A. , Clark , J. C. , Adams , L. , Guz , A. , & Frackowiak , R. S. ( 1993 ). Changes in global cerebral blood flow in humans: Effect on regional cerebral blood flow during a neural activation task . The Journal of Physiology , 471 ( 1 ), 521 – 534 . 10.1113/jphysiol.1993.sp019913 8120819 PMC1143974

[b102] Ratnatunga , C. , & Adiseshiah , M. ( 1990 ). Increase in middle cerebral artery velocity on breath holding: A simplified test of cerebral perfusion reserve . European Journal of Vascular Surgery , 4 ( 5 ), 519 – 523 . 10.1016/S0950-821X(05)80795-9 2121547

[b103] Ravi , H. , Liu , P. , Peng , S.-L. , & Lu , H. ( 2015 ). Multiband BOLD acquisition enhances the sensitivity of cerebrovascular reactivity (CVR) mapping . 10.1002/nbm.3600

[b104] Ravi , H. , Thomas , B. P. , Peng , S.-L. , Liu , H. , & Lu , H. ( 2016 ). On the optimization of imaging protocol for the mapping of cerebrovascular reactivity . Journal of Magnetic Resonance Imaging , 43 ( 3 ), 661 – 668 . 10.1002/jmri.25028 26268541 PMC4752936

[b105] Sasse , S. A. , Berry , R. B. , Nguyen , T. K. , Light , R. W. , & Mahutte , C. K. ( 1996 ). Arterial blood gas changes during breath-holding from functional residual capacity . Chest , 110 ( 4 ), 958 – 964 . 10.1378/chest.110.4.958 8874252

[b106] Sato , K. , Sadamoto , T. , Hirasawa , A. , Oue , A. , Subudhi , A. W. , Miyazawa , T. , & Ogoh , S. ( 2012 ). Differential blood flow responses to CO2 in human internal and external carotid and vertebral arteries . The Journal of Physiology , 590 ( Pt 14 ), 3277 – 3290 . 10.1113/jphysiol.2012.230425 22526884 PMC3459042

[b107] Sayin , E. S. , Schulman , J. , Poublanc , J. , Levine , H. T. , Raghavan , L. V. , Uludag , K. , Duffin , J. , Fisher , J. A. , Mikulis , D. J. , & Sobczyk , O. ( 2023 ). Investigations of hypoxia-induced deoxyhemoglobin as a contrast agent for cerebral perfusion imaging . Human Brain Mapping , 44 ( 3 ), 1019 – 1029 . 10.1002/hbm.26131 36308389 PMC9875930

[b108] Sayin , E. S. , Sobczyk , O. , Poublanc , J. , Mikulis , D. J. , Fisher , J. A. , Kuo , K. H. M. , & Duffin , J. ( 2022 ). Assessment of cerebrovascular function in patients with sickle cell disease using transfer function analysis . Physiological Reports , 10 ( 19 ), e15472 . 10.14814/phy2.15472 36200271 PMC9535348

[b109] Schulman , J. B. , Kashyap , S. , Kim , S. G. , & Uludağ , K. ( 2024 ). Non-invasive perfusion MR imaging of the human brain via breath-holding . Scientific Reports , 14 ( 1 ), 7322 . 10.1038/s41598-024-58086-8 38538842 PMC10973507

[b204] Schulman , J. , Sayin , S. , Poublanc , J. , Manalac , A. , Fisher , J. , Sobczyk, O., Duffin, J., Levine, H., Mikulis, D., & Uludag, K. ( 2022 ). Deoxyhemoglobin versus Gadolinium as Contrast in Dynamic Susceptibility Perfusion Imaging: Simulations and Scan Validations . ISMRM , London, England .

[b110] Schulman , J. B. , Sayin , E. S. , Manalac , A. , Poublanc , J. , Sobczyk , O. , Duffin , J. , Fisher , J. A. , Mikulis , D. , & Uludağ , K. ( 2023 ). DSC MRI in the human brain using deoxyhemoglobin and gadolinium-Simulations and validations at 3T . Frontiers in Neuroimaging , 2 , 1048652 . 10.3389/fnimg.2023.1048652 37554650 PMC10406263

[b111] Severinghaus , J. W. ( 1979 ). Simple, accurate equations for human blood O2 dissociation computations . Journal of Applied Physiology: Respiratory, Environmental and Exercise Physiology , 46 ( 3 ), 599 – 602 . 10.1152/jappl.1979.46.3.599 35496

[b112] Sleight , E. , Stringer , M. S. , Marshall , I. , Wardlaw , J. M. , & Thrippleton , M. J. ( 2021 ). Cerebrovascular reactivity measurement using magnetic resonance imaging: A systematic review . Frontiers in Physiology , 12 , 643468 . 10.3389/fphys.2021.643468 33716793 PMC7947694

[b113] Sleight , E. , Stringer , M. S. , Mitchell , I. , Murphy , M. , Marshall , I. , Wardlaw , J. M. , & Thrippleton , M. J. ( 2023 ). Cerebrovascular reactivity measurements using 3T BOLD MRI and a fixed inhaled CO2 gas challenge: Repeatability and impact of processing strategy . Frontiers in Physiology , 14 , 1070233 . 10.3389/fphys.2023.1070233 36814481 PMC9939770

[b115] Smitha , K. , Akhil Raja , K. , Arun , K. , Rajesh , P. , Thomas , B. , Kapilamoorthy , T. , & Kesavadas , C. ( 2017 ). Resting state fMRI: A review on methods in resting state connectivity analysis and resting state networks . The Neuroradiology Journal , 30 ( 4 ), 305 – 317 . 10.1177/1971400917697342 28353416 PMC5524274

[b116] Sobczyk , O. , Battisti-Charbonney , A. , Fierstra , J. , Mandell , D. M. , Poublanc , J. , Crawley , A. P. , Mikulis , D. J. , Duffin , J. , & Fisher , J. A. ( 2014 ). A conceptual model for CO _2_ -induced redistribution of cerebral blood flow with experimental confirmation using BOLD MRI . NeuroImage , 92 , 56 – 68 . 10.1016/j.neuroimage.2014.01.051 24508647

[b117] Sobczyk , O. , Crawley , A. P. , Poublanc , J. , Sam , K. , Mandell , D. M. , & Mikulis , D. J. ( 2016 ). Identifying significant changes in cerebrovascular reactivity to carbon dioxide . AJNR: American Journal of Neuroradiology , 37 , 818 – 824 . 10.3174/ajnr.A4679 26846924 PMC7960311

[b118] Sobczyk , O. , Sam , K. , Mandell , D. M. , Crawley , A. P. , Venkatraghavan , L. , McKetton , L. , Poublanc , J. , Duffin , J. , Fisher , J. A. , & Mikulis , D. J. ( 2020 ). Cerebrovascular reactivity assays collateral function in carotid stenosis . Frontiers in Physiology , 11 , 1031 . 10.3389/fphys.2020.01031 33041841 PMC7528398

[b119] Sobczyk , O. , Sayin , E. S. , Sam , K. , Poublanc , J. , Duffin , J. , Fisher , J. A. , & Mikulis , D. J. ( 2021 ). The reproducibility of cerebrovascular reactivity across MRI scanners . Frontiers in Physiology , 12 , 668662 . 10.3389/fphys.2021.668662 34025455 PMC8134667

[b120] Stefanovic , B. , Warnking , J. M. , Rylander , K. M. , & Pike , G. B. ( 2006 ). The effect of global cerebral vasodilation on focal activation hemodynamics . NeuroImage , 30 ( 3 ), 726 – 734 . 10.1016/j.neuroimage.2005.10.038 16337135

[b121] Stickland , R. C. , Zvolanek , K. M. , Moia , S. , Ayyagari , A. , Caballero-Gaudes , C. , & Bright , M. G. ( 2021 ). A practical modification to a resting state fMRI protocol for improved characterization of cerebrovascular function . NeuroImage , 239 , 118306 . 10.1016/j.neuroimage.2021.118306 34175427 PMC8552969

[b122] Stickland , R. C. , Zvolanek , K. M. , Moia , S. , Caballero-Gaudes , C. , & Bright , M. G. ( 2022 ). Lag-optimized blood oxygenation level dependent cerebrovascular reactivity estimates derived from breathing task data have a stronger relationship with baseline cerebral blood flow . Frontiers in Neuroscience , 16 , 910025 . 10.3389/fnins.2022.910025 35801183 PMC9254683

[b123] Sur , S. , Lin , Z. , Li , Y. , Yasar , S. , Rosenberg , P. B. , Moghekar , A. , Agarwal , S. , Hou , X. , Jiang , D. , Kalyani , R. , Hazel , K. , Pottanat , G. , Xu , C. , Van Zijl , P. , Pillai , J. , Liu , P. , Albert , M. S. , & Lu , H. ( 2020 ). CO2 cerebrovascular reactivity measured with phase‐contrast MRI: A potential biomarker of cognition and physical function in older adults . Alzheimer’s & Dementia the Journal of the Alzheimer’s Association , 16 ( S4 ), e042215 . https://alz-journals.onlinelibrary.wiley.com/doi/full/10.1002/alz.042215

[b124] Symon , L. ( 1968 ). Experimental evidence for “intracerebral steal” following CO2 inhalation . Scandinavian Journal of Clinical and Laboratory Investigation , 21 ( Suppl. 102 ), XIII:A . 10.3109/00365516809169044 4974242

[b125] Tancredi , F. B. , Gauthier , C. J. , Madjar , C. , Bolar , D. S. , Fisher , J. A. , Wang , D. J. J. , & Hoge , R. D. ( 2012 ). Comparison of pulsed and pseudocontinuous arterial spin-labeling for measuring CO2-induced cerebrovascular reactivity . Journal of Magnetic Resonance Imaging , 36 ( 2 ), 312 – 321 . 10.1002/jmri.23658 22544711

[b126] Tancredi , F. B. , & Hoge , R. D. ( 2013 ). Comparison of cerebral vascular reactivity measures obtained using breath-holding and CO2 inhalation . Journal of Cerebral Blood Flow and Metabolism: Official Journal of the International Society of Cerebral Blood Flow and Metabolism , 33 ( 7 ), 1066 – 1074 . 10.1038/jcbfm.2013.48 23571282 PMC3705433

[b127] Taneja , K. , Liu , P. , Xu , C. , Turner , M. , Zhao , Y. , Abdelkarim , D. , Thomas , B. P. , Rypma , B. , & Lu , H. ( 2020 ). Quantitative cerebrovascular reactivity in normal aging: comparison between phase-contrast and arterial spin labeling MRI . Frontiers in Neurology , 11 , 758 . 10.3389/fneur.2020.00758 32849217 PMC7411174

[b128] Thomas , B. P. , Liu , P. , Aslan , S. , King , K. S. , Osch , M. J. P. , & Lu , H. ( 2013 ). Physiologic underpinnings of negative BOLD cerebrovascular reactivity in brain ventricles . NeuroImage , 83 , 505 – 512 . 10.1016/j.neuroimage.2013.07.005 23851322 PMC3815980

[b129] Triantafyllou , C. , Wald , L. L. , & Hoge , R. D. ( 2011 ). Echo-time and field strength dependence of BOLD reactivity in veins and parenchyma using flow-normalized hypercapnic manipulation . PLoS One , 6 ( 9 ), e24519 . 10.1371/journal.pone.0024519 21915346 PMC3167856

[b130] Uludağ , K. ( 2010 ). To dip or not to dip: Reconciling optical imaging and fMRI data . Proceedings of the National Academy of Sciences of the United States of America , 107 , E23 . 10.1073/pnas.0914194107 20142469 PMC2823892

[b131] Uludağ , K. , & Blinder , P. ( 2018 ). Linking brain vascular physiology to hemodynamic response in ultra-high field MRI . NeuroImage , 168 , 279 – 295 . 10.1016/j.neuroimage.2017.02.063 28254456

[b132] Uludağ , K. , Müller-Bierl , B. , & Ugurbil , K. ( 2009 ). An integrative model for neuronal activity-induced signal changes for gradient and spin echo functional imaging . NeuroImage , 48 , 150 – 165 . 10.1016/j.neuroimage.2009.05.051 19481163

[b133] van den Heuvel , M. P. , & Hulshoff Pol , H. E . ( 2010 ). Exploring the brain network: A review on resting-state fMRI functional connectivity . European Neuropsychopharmacology: The Journal of the European College of Neuropsychopharmacology , 20 ( 8 ), 519 – 534 . 10.1016/j.euroneuro.2010.03.008 20471808

[b134] van Niftrik , C. H. B. , Piccirelli , M. , Bozinov , O. , Maldaner , N. , Strittmatter , C. , Pangalu , A. , Valavanis , A. , Regli , L. , & Fierstra , J. ( 2018 ). Impact of baseline CO2 on Blood-Oxygenation-Level-Dependent MRI measurements of cerebrovascular reactivity and task-evoked signal activation . Magnetic Resonance Imaging , 49 , 123 – 130 . 10.1016/j.mri.2018.02.002 29447850

[b135] Venkatraghavan , L. , Poublanc , J. , Han , J. S. , Sobczyk , O. , Rozen , C. , Sam , K. , Duffin , J. , Mikulis , D. J. , & Fisher , J. A. ( 2018 ). Measurement of cerebrovascular reactivity as blood oxygen level-dependent magnetic resonance imaging signal response to a hypercapnic stimulus in mechanically ventilated patients . Journal of Stroke and Cerebrovascular Diseases , 27 ( 2 ), 301 – 308 . 10.1016/j.jstrokecerebrovasdis.2017.08.035 28967593

[b136] Vesely , A. , Sasano , H. , Volgyesi , G. , Somogyi , R. , Tesler , J. , Fedorko , L. , Grynspan , J. , Crawley , A. , Fisher , J. A. , & Mikulis , D. ( 2001 ). MRI mapping of cerebrovascular reactivity using square wave changes in end-tidal PCO2 . Magnetic Resonance in Medicine , 45 ( 6 ), 1011 – 1013 . 10.1002/mrm.1134 11378878

[b137] Vestergaard , M. B. , & Larsson , H. B. ( 2019 ). Cerebral metabolism and vascular reactivity during breath-hold and hypoxic challenge in freedivers and healthy controls . Journal of Cerebral Blood Flow & Metabolism , 39 ( 5 ), 834 – 848 . 10.1177/0271678X17737909 29099292 PMC6498754

[b138] Vovenko , E. ( 1999 ). Distribution of oxygen tension on the surface of arterioles, capillaries and venules of brain cortex and in tissue in normoxia: An experimental study on rats . Pflugers Archiv , 437 , 617 – 623 . 10.1007/s004240050825 10089576

[b205] Vu , C. , Bush , A. , Choi , S. , Miao , X. , Coates , T. D. , & Wood , J. C . ( 2017 ). Chronic anemia is associated with lower cerebral and peripheral arterio-venous oxygen gradients . Blood , 130 , 3542 . https://www.sciencedirect.com/science/article/pii/S0006497119840582

[b139] Vu , C. , Chai , Y. , Coloigner , J. , Nederveen , A. J. , Borzage , M. , Bush , A. , & Wood , J. C. ( 2021 ). Quantitative perfusion mapping with induced transient hypoxia using BOLD MRI . Magnetic Resonance in Medicine , 85 ( 1 ), 168 – 181 . 10.1002/mrm.28422 32767413 PMC7728454

[b140] Vu , C. , Xu , B. , González-Zacarías , C. , Shen , J. , Baas , K. , Choi , S. , Nederveen , A. , & Wood , J. ( 2023 ). Sinusoidal CO2 respiratory challenge for concurrent perfusion and cerebrovascular reactivity MRI . Frontiers in Physiology , 14 , 1102983 . 10.3389/fphys.2023.1102983 36846345 PMC9948030

[b141] Waddle , S. L. , Juttukonda , M. R. , Lants , S. K. , Davis , L. T. , Chitale , R. , Fusco , M. R. , Jordan , L. C. , & Donahue , M. J. ( 2020 ). Classifying intracranial stenosis disease severity from functional MRI data using machine learning . Journal of Cerebral Blood Flow & Metabolism , 40 ( 4 ), 705 – 719 . 10.1177/0271678X19848098 31068081 PMC7168799

[b142] Wang , P. , Hou , P. , Kesler , S. , Colen , R. , Kumar , A. , Prabhu , S. , & Liu , H. ( 2016 ). SU-G-IeP1-11: Resting-state fluctuation of BOLD signal amplitude for mapping cerebrovascular reactivity in presurgical functional MRI . Medical Physics , 43 ( 6Part25 ), 3646 – 3647 . 10.1118/1.4956971

[b143] Wise , R. G. , Ide , K. , Poulin , M. J. , & Tracey , I. ( 2004 ). Resting fluctuations in arterial carbon dioxide induce significant low frequency variations in BOLD signal . NeuroImage , 21 ( 4 ), 1652 – 1664 . 10.1016/j.neuroimage.2003.11.025 15050588

[b144] Xu , F. , Xu , C. , Zhu , D. , Liu , D. , Lu , H. , & Qin , Q. ( 2024 ). Evaluating cerebrovascular reactivity measured by velocity selective inversion arterial spin labeling with different post-labeling delays: The effect of fast flow . Magnetic Resonance in Medicine , 92 ( 5 ), 2065 – 2073 . 10.1002/mrm.30166 38852173 PMC11341248

[b145] Yablonskiy , D. A. , & Haacke , E. M. ( 1994 ). Theory of NMR signal behavior in magnetically inhomogeneous tissues: The static dephasing regime . Magnetic Resonance in Medicine , 32 ( 6 ), 749 – 763 . 10.1002/mrm.1910320610 7869897

[b146] Yezhuvath , U. S. , Uh , J. , Cheng , Y. , Martin-Cook , K. , Weiner , M. , Diaz-Arrastia , R. , van Osch , M. , & Lu , H. ( 2012 ). Forebrain-dominant deficit in cerebrovascular reactivity in Alzheimer’s disease . Neurobiology of Aging , 33 ( 1 ), 75 – 82 . 10.1016/j.neurobiolaging.2010.02.005 20359779 PMC2896562

[b147] Zang , Y.-F. , He , Y. , Zhu , C.-Z. , Cao , Q.-J. , Sui , M.-Q. , Liang , M. , Tian , L.-X. , Jiang , T.-Z. , & Wang , Y.-F. ( 2007 ). Altered baseline brain activity in children with ADHD revealed by resting-state functional MRI . Brain & Development , 29 ( 2 ), 83 – 91 . 10.1016/j.braindev.2006.07.002 16919409

[b148] Zappe , A. C. , Uludağ , K. , Oeltermann , A. , Uğurbil , K. , & Logothetis , N. K. ( 2008 ). The influence of moderate hypercapnia on neural activity in the anesthetized nonhuman primate . Cerebral Cortex (New York, N.Y.: 1991) , 18 ( 11 ), 2666 – 2673 . 10.1093/cercor/bhn023 18326521 PMC2567427

[b206] Zhao , J. M. , Clingman , C. S. , Närväinen , M. J. , Kauppinen , R. A. , & van Zijl , P. C. M. ( 2007 ). Oxygenation and hematocrit dependence of transverse relaxation rates of blood at 3T . Magnetic Resonance in Medicine , 58 ( 3 ), 592 – 597 . 10.1002/mrm.21342 17763354

[b149] Zhao , M. Y. , Fan , A. P. , Chen , D. Y.-T. , Sokolska , M. J. , Guo , J. , Ishii , Y. , Shin , D. D. , Khalighi , M. M. , Holley , D. , Halbert , K. , Otte , A. , Williams , B. , Rostami , T. , Park , J.-H. , Shen , B. , & Zaharchuk , G. ( 2021 ). Cerebrovascular reactivity measurements using simultaneous 15O-water PET and ASL MRI: Impacts of arterial transit time, labeling efficiency, and hematocrit . NeuroImage , 233 , 117955 . 10.1016/j.neuroimage.2021.117955 33716155 PMC8272558

[b150] Zhou , Y. , Rodgers , Z. B. , & Kuo , A. H. ( 2015 ). Cerebrovascular reactivity measured with arterial spin labeling and blood oxygen level dependent techniques . Magnetic Resonance Imaging , 33 ( 5 ), 566 – 576 . 10.1016/j.mri.2015.02.018 25708263 PMC4426232

[b151] Zou , Q.-H. , Zhu , C.-Z. , Yang , Y. , Zuo , X.-N. , Long , X.-Y. , Cao , Q.-J. , Wang , Y.-F. , & Zang , Y.-F. ( 2008 ). An improved approach to detection of amplitude of low-frequency fluctuation (ALFF) for resting-state fMRI: Fractional ALFF . Journal of Neuroscience Methods , 172 ( 1 ), 137 – 141 . 10.1016/j.jneumeth.2008.04.012 18501969 PMC3902859

[b152] Zuo , X.-N. , Di Martino , A. , Kelly , C. , Shehzad , Z. E. , Gee , D. G. , Klein , D. F. , Castellanos , F. X. , Biswal , B. B. , & Milham , M. P. ( 2010 ). The oscillating brain: Complex and reliable . NeuroImage , 49 ( 2 ), 1432 – 1445 . 10.1016/j.neuroimage.2009.09.037 19782143 PMC2856476

