# Supplementary material for: Problems and solutions in quantifying cerebrovascular reactivity using BOLD-MRI
Source: Imaging Neurosci (Camb). 2025 May 2;3:imag_a_00556. doi: 10.1162/imag_a_00556 (PMC12319970; doi:10.1162/imag_a_00556)
Supplement: Supplementary Material [file imag_a_00556-supp.pdf]

1 Experimental Methods

1.1 Subjects

This study was approved by the Research Ethics Board of Sungkyunkwan University and all procedures followed the principles expressed in the Declaration of Helsinki. Informed consent was obtained in all 10 healthy volunteers (age: 30.4 ± 9.4 years, 3 female). Please note that the same subjects were scanned at both 3 T and 7 T. One subject was excluded from group analysis due to an error in the multi-band calibration.

1.2 MRI Sequences and Experimental Protocols

MRI data were acquired at the Center for Neuroscience Imaging Research at Sungkyunkwan University on the Siemens 3 T Prisma (Siemens Healthineers, Erlangen, Germany) and Siemens 7 T Terra (Siemens Healthineers, Erlangen, Germany) using the commercially available 64 channel head/neck and 32 channel head coils (Nova Medical, Wilmington, USA), respectively.

Structural MRI

The 3 T parameters were as follows: 3D-MP2RAGE (Marquez *et al.,* 2010) with 1.0 mm isotropic spatial resolution (176 sagittal slices; GRAPPA = 3; Ref lines PE = 32; FoVread = 250 mm; phase-encoding = A-P; TI1/TI2 = 700/2500 ms; α1/α2 = 4°/5°; TE/TR = 2.98/5000 ms; bandwidth = 240 Hz/px; echo-spacing = 7.1 ms; TA = 8:22 min).

The 7 T parameters were as follows: 3D-MP2RAGE (Marquez *et al.,* 2010) with 0.7 mm isotropic spatial resolution (240 sagittal slices; GRAPPA = 3; Ref lines PE = 36; FoVread = 224 mm; phase-encoding = A-P; TI1/TI2 = 1000/3200 ms; α1/α2 = 4°/4°; TE/TR = 2.29/4500 ms; partial-$\text{Fourier}_{\text{slice}}$ = 6/8; bandwidth = 200 Hz/px; echo-spacing = 7.3 ms; TA = 9:15 min).

Resting-State MRI

The 3 T parameters were as follows: Gradient-echo 2D-EPI (GRE-EPI) with 2.0 mm isotropic spatial resolution (64 interleaved axial slices; GRAPPA = 2; Ref lines PE = 46; SMS = 2; Ref. scan = EPI; FatSat = True; FoVread = 192 mm; phase-encoding = P-A; TE = 30 ms; α = 70°; TR = 2000 ms; bandwidth = 2312 Hz/px; echo-spacing = 0.53 ms; EPI factor = 94) was acquired for the resting-state experiment (607 TRs, TA = 20:14 min). 6 measurements of opposite phase-encoded (A-P) data were also acquired for distortion correction.

The 7 T parameters were as follows: GRE-EPI with 2.0 mm isotropic spatial resolution (74 interleaved axial slices; GRAPPA = 3; Ref lines PE = 54; SMS = 2; Ref. scan = EPI; partial-$\text{Fourier}_{\text{phase}}$ = 6/8; FatSat = True; FoVread = 192 mm; phase-encoding = P-A; TE = 18 ms; α = 50°; TR = 2000 ms; bandwidth = 2368 Hz/px; echo-spacing = 0.53 ms; EPI factor = 96) was acquired for the resting-state experiment (613 TRs, TA = 20:26 min). 6 measurements of opposite phase-encoded (A-P) data were also acquired for distortion correction. Please note that the spatial resolution has **not** been optimized for 7 T in this study. We have instead chosen the same spatial resolution as at 3 T to allow for easier and direct comparisons.

For the resting-state scan, no instructions were provided to the subjects other than to rest.

Breath-hold MRI

The breath-hold and resting-state acquisition parameters were identical, except for the acquisition time (Breath-holding: 260 TRs and TA = 8:53 min at 3 T; 250 TRs and TA = 8:46 min at 7 T).

For the breath-hold scans, subjects were instructed to fixate on a black cross at the center of an iso-luminant gray screen and a countdown timer, which indicated when to breathe regularly and when to perform a breath-hold. Specifically, each breath-hold block was composed of three sections: 10 s of preparation with regular breathing, 16 s of breath-holding, and 34 s of regular breathing (Schulman *et al.*, 2024). This was repeated nine times to obtain multiple boluses, with an added final baseline of 80 s. Only the first eight boluses were used for each subject as the response to the final breath-hold bolus was not fully included during the acquisition for some subjects.

1.3 Preprocessing

FSL (version 6.0.4), AFNI (version 23.0.07), and in-house Python scripts (https://github.com/JSchul1998/bhDSC_Scripts) were used for image pre-processing (Cox, 1996; Jenkinson *et al.*, 2012). Anatomical, resting-state, and breath-hold T_2^* data from both scanners were corrected for gradient nonlinearities using the Human Connectome Project's version of the gradunwarp tool (https://github.com/Washington-University/gradunwarp).

The MP2RAGE structural data were pre-processed using presurfer (https://github.com/srikash/presurfer) and skull-stripped using SynthStrip (Hoopes *et al.*, 2022). The background denoised T1-weighted UNI image was segmented using FSL FAST (Zhang *et al.*, 2001) to obtain three tissue classes corresponding to gray matter (GM), white matter (WM), and cerebrospinal fluid (CSF). The resting-state and breath-hold data underwent slice-timing correction (3dTshift, AFNI), motion correction (mcflirt, FSL), distortion correction (topup, FSL), and skull stripping (SynthStrip) (Andersson *et al.*, 2003; Hoopes *et al.*, 2022; Jenkinson *et al.*, 2002). The resulting breath-hold data were then linearly detrended and temporally filtered by averaging each signal time point with a 1x5 Gaussian kernel.

Pre-processed breath-hold and resting-state signal time courses ($S(t)$) were then converted to relaxation rate time courses ($\Delta R_2^*(t)$) at 3 T (TE = 0.03 s) and 7 T (TE = 0.018 s).

For the resting-state data, the baseline signal (S_0) was defined as the average of the full time course; for the breath-hold data, S_0 was defined as the average signal of 10 temporal volumes before and after each breath-hold bolus.

1.4 Gray and White Matter Segmentation

GM and WM masks were generated, at both 3 T and 7 T, using whole brain anatomical data (FSL FAST). The partial volume estimate maps were then thresholded at 0.9, binarized, and transformed from anatomical to GRE-EPI space for each subject (nearest-neighbor interpolation). Arterial and venous voxels were removed from the masks by thresholding a GRE-EPI temporal standard deviation (ε_t) map (only voxels with values in the lowest 10% of the ε_t range were kept in the mask) and using the output to mask the binarized FAST segmentations—the assumption being that voxels with higher blood volume have higher physiological noise due to pulsatility, leading to higher values of ε_t (Schulman *et al.*, 2023; Kashyap *et al.*, 2018).

1.5 Defining an Arterial Input Regressor

While CVR_{BOLD} regression studies often implement $P_{ET}CO_2(t)$ as an input regressor (Pinto *et al.*, 2021), we did not collect these physiological data. Instead, using a recently developed method to extract an arterial vasodilatory time course in response to hypercapnia (Schulman *et al.*, 2024), we implemented a novel, equipment-free, input regressor for both breath-hold and resting-state paradigms. Importantly, by using an arterial time course as an input regressor, the physiological delay and dispersion attributed to $P_{ET}CO_2$ is mitigated. We have updated our approach for creating the arterial input mask:

1) A general arterial ROI of the axial slices containing the middle (MCA), posterior (PCA), and anterior (ACA) cerebral arteries is first identified based on the MP2RAGE structural data.
2) Resting-state time courses within the arterial ROI undergo Butterworth filtering from 0.0075 to 0.075 Hz (removing low and high frequency fluctuations outside of the typical frequency range of CO_2 fluctuations); this step is not conducted for the breath-hold data.
3) Each voxel within the arterial ROI is vertically flipped (i.e., to account for the fact that arterial vasodilation leads to a signal decrease, while increased tissue OHb yields a signal increase).
4) A whole brain (GM and WM) average time course is calculated (high-pass filtered at 0.0075 Hz for resting-state data).
5) Each voxel within the arterial ROI is shifted forward and backward in 1 TR (2 s) steps, up to a maximum of 8 s forward and 8 s backward, and the correlation between the candidate arterial time course and whole brain average time course is recorded for each shift. The temporal shift resulting in the highest correlation is determined to be the delay in a model-independent manner. A similar delay method has been employed in previous studies using resting-state fMRI data (Khalil *et al.*, 2017; Lv *et al.*, 2013).
6) Voxels within the arterial ROI that have a correlation at or above the 99th percentile and a delay/onset prior to tissue (by 2 s or more) are included in the arterial mask.
7) The arterial mask is then visually inspected to remove any voxels outside of the MCA, PCA, or ACA, resulting in the final arterial regressor mask.

1.6 CVR Quantification

The resting-state data were temporally filtered within the range of 0.01-0.045 Hz (i.e., roughly within the frequency range that best correlates with endogenous CO_2 fluctuations (Liu *et al.*, 2017; Wise *et al.*, 2004)). For both resting-state and breath-hold paradigms, the relaxation rate time courses were linearly interpolated to a temporal resolution of 0.5 s. Time courses within the arterial regressor mask were vertically flipped and averaged voxel-wise, to generate the arterial input regressor function (AIF). The AIF was fit to each tissue voxel by first shifting the AIF forward in 0.5 s steps, up to a maximum of 7 s, and the correlation between the AIF and tissue time courses were recorded for each shift. The temporal shift resulting in the highest correlation was recorded as the voxel's **delay** (d); the associated **correlation** was recorded as well. The AIF was then fit to the time course in each voxel ($Tissue(t)$), using the voxel's delay as an input parameter, assuming the following model:

$$Tissue(t) = \left(CVR^*_{BOLD,R}\right) \cdot AIF(t-d) + s + \varepsilon(t) \qquad \text{(S1)}$$

Here, $CVR^*_{BOLD,R}$ is effectively the vertical stretch that minimizes least squared error between the tissue and AIF relaxation rate time courses, and is a proxy to $CVR_{BOLD,R}$ (while absolute scaling will be different from $CVR_{BOLD,R}$, we do not expect regional scaling differences, which is the only measure of concern in this work). $CVR^*_{BOLD,R}$ was further normalized to the average GM $CVR^*_{BOLD,R}$ to obtain $\boldsymbol{CVR}^*$ maps. s represents the vertical shift needed to optimize the fitting and $\varepsilon(t)$ represents the residual error.

Note that we also attempted to use an average GM input regressor; we found the GM regressor correlated similarly to the AIF, with the only limitation being that delay maps were not absolute (i.e., delay was either prior to or after the average GM time course); thus, we opted to use the AIF.

To facilitate group-level analysis, the CVR^*, delay, and correlation maps were transformed to FSL's MNI152 2 mm space. The processed data were first registered to the subject's anatomical space (6 dof, FSL flirt) and the anatomical data were then non-linearly registered (fnirt, FSL) to the FSL MNI152 2 mm template (Jenkinson *et al.,* 2012; Jenkinson *et al.,* 2001). The two transformation matrices were then combined into a subject-specific native-to-MNI warp and applied to all subject-specific breath-hold and resting-state maps. The MNI space-transformed maps were then averaged across all subjects to generate mean CVR^*, delay, and correlation maps.

1.7 Statistics

We found the subject-averaged CVR^* data to be normally distributed using the Shapiro-Wilk test. Thereafter, we performed a two-tailed paired voxel-wise t-test ($\propto = 0.05$) to compare breath-hold and resting-state CVR^* at both 3 T and 7 T.

2 Simulation Methods

2.1 Disease Modeling: Vascular Occlusion

The vascular occlusion simulation follows that of the general simulation model described in Section 2.2 of the main paper, with three major modifications.

First, as opposed to setting ΔCBF_p to 40%, we simulated ΔCBF_p as a time course (scaled by 40%):

$$\Delta CBF_p(t) = \left[40 \cdot \left(\frac{t}{3}\right)^3 \cdot e^{\left(3\cdot\left(1-\frac{t}{3}\right)\right)}\right] * R(t) \qquad \text{(S2.1)}$$

$$R(t) = \frac{e^{\left(-\left(\frac{t-d}{D}\right)\right)}}{D} \qquad \text{(S2.2)}$$

Here, a simple gamma-variate function was simulated to roughly model the time course duration of a 16 s breath-hold bolus (Schulman *et al.,* 2024). Additionally, to capitulate the effects of stenosis-induced delay and dispersion, the gamma-variate was convolved with a dispersion function ($R(t)$), parameterized with delay (*d*, in seconds) and dispersion (*D*, in seconds). In the scenario of no occlusion (and for the collateral vessel; see below), this convolution was not performed.

Second, we modeled a tissue voxel supplied by two independent arterial vessels.

Vessel A was modeled with a variable degree of stenosis, ranging in baseline flow from 0% (i.e., no measured signal change in tissue) to 100% (i.e., measured signal change in tissue the same as the general simulations). Thus, the simulated $\Delta CBF_p(t)$ bolus entering tissue from vessel A was scaled down in magnitude and dispersed/delayed depending on the degree of baseline flow reduction (e.g., if flow was reduced by 50%, so was the input $\Delta CBF_p(t)$ bolus magnitude, and it was delayed/dispersed by 2 seconds

(assuming typical transit time and dispersion from artery to tissue at 100% baseline flow is ~ 1-3 seconds (Ibaraki *et al.,* 2007; Schulman *et al.,* 2024))).

Vessel B was modeled to supply tissue with a variable degree of collateral flow, ranging in baseline flow from 0% (i.e., no collateral flow) to 100% (i.e., collateral flow equal to 100% of flow supplied by a non-stenotic vessel A) (Figure 8A). Thus, the simulated $\Delta CBF_p(t)$ bolus entering tissue from vessel B was scaled up in magnitude depending on the degree of baseline flow with respect to non-stenotic vessel A (e.g., if vessel B flow = 50% of non-stenotic vessel A flow, then vessel B $\Delta CBF_p(t)$ input bolus magnitude = 50% of vessel A $\Delta CBF_p(t)$ input bolus magnitude). The $\Delta CBF_p(t)$ input time courses from vessels A and B were then summated and served as the tissue $\Delta CBF_p(t)$ bolus for all calculations (refer to Section 2.2 of main paper).

Third, the tissue microvasculature was simulated to compensate for flow changes in the upstream vasculature (i.e., autoregulation) if the cumulative upstream flow (i.e., summated flow from vessels A and B) was greater than at least half of the baseline flow in non-stenotic vessel A (Lassen *et al.,* 1959). If baseline flow was lower than this value, tissue vasculature CBV was made to be unresponsive to any further reduction in flow and unresponsive to the vasodilatory stimulus (as the tissue microvasculature will have reached vasodilatory capacity).

2.2 Disease Modeling: Steal Physiology

Please note that the steal physiology simulations are independent of the simulations described in Section 2.2 of the manuscript. Here, modeling was performed as a parallel flow circuit. Baseline resistance in Vessels 2 (R_2) and 3 (R_3) was set to 1; baseline resistance in Vessels 1 (R_1) and 4 (R_4), which represents a summation of vasculature prior to and after the parallel circuit, respectively (see Supplementary Figure 7), was set to 1/3. Although an estimation, the resistance of 1/3 was determined given the assumption that vessels prior to (and after) the parallel circuit were composed of vessels doubling in CBV (and thus, quartering in resistance)—1/3 is the infinite sum of resistance in this series. The resistance of 1 for Vessels 2 and 3 was arbitrarily chosen as its absolute value does not affect the simulations so long as the 1:1/3 relationship is preserved.

As "voltage" (V) was not found to affect the quantitative values in this simulation, it was set to 1. With the circuit parameters established, Kirchoff's laws were then used to calculate the corresponding flow through Vessel 3 (I_3), in accordance with Eq. S3:

$$I_3 = \frac{V}{R_3} \cdot \left(1 - \frac{(R_1 + R_4)}{\left(R_1 + R_4 + \left(\frac{R_2 \cdot R_3}{R_2 + R_3} \right) \right)} \right) \qquad \text{(S3)}$$

This flow calculation (I_3) was iterated by reducing resistance (analogous to adding vasodilatory agent) from 0-100% in Vessels 2 and 3 (x- and y-axis respectively in Figure 9B). We assume that Vessel 4 (i.e., venous vasculature) sees a negligible resistance change relative to the other vessels (Grubb et al., 1974; Ito *et al.,* 2003) and Vessel 1, due to simulated occlusion, also sees a negligible change—for the scenario where Vessel 1 is capable of vasodilation, see Supplementary Figure 8.

3 Supplementary Figures

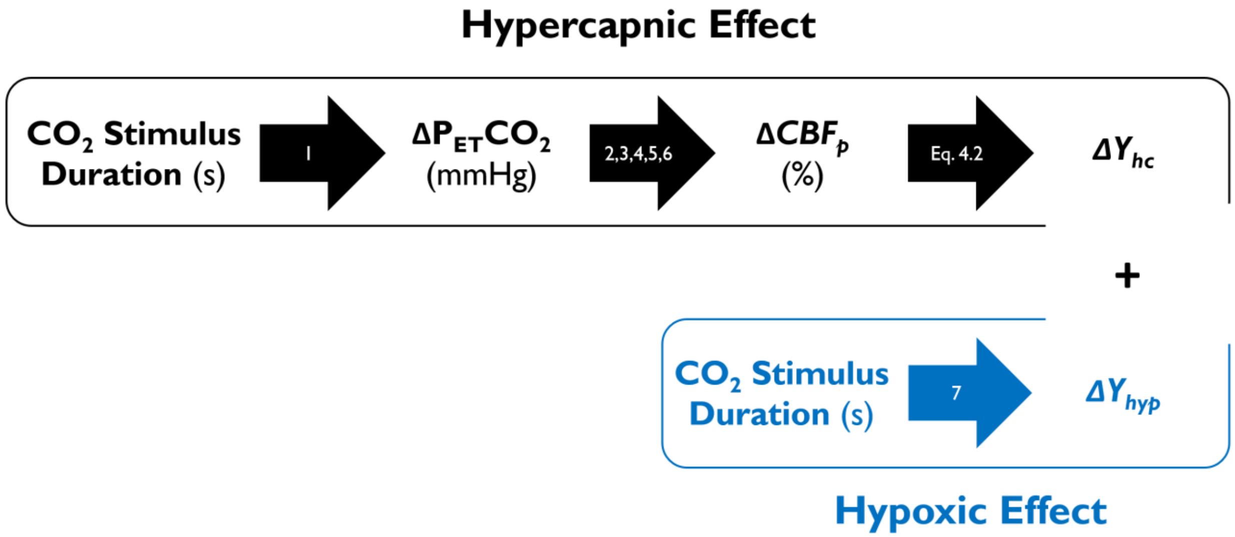

Supplementary Figure 1. Flow Chart to Illustrate Breath-Hold Simulations. *[1]In accordance with Figure 4 in Sasse et al., 1996. [2,3,4,5,6]Relationships relating $\Delta P_{ET}CO_2$ to ΔCBF_p shown in Grune et al., 2015; Poulin et al., 1996; Ramsay et al., 1993; Sato et al., 2012; Tancredi and Hoge, 2013. [7]In accordance with Figure 2 in Sasse et al., 1996 and then converted from P_aO_2 to ΔY_{hyp} using equations in Severinghaus, 1979. Steps in blue are only performed for breath-hold simulations with hypoxia (not without hypoxia (i.e., gas-control-induced hypercapnia)).*

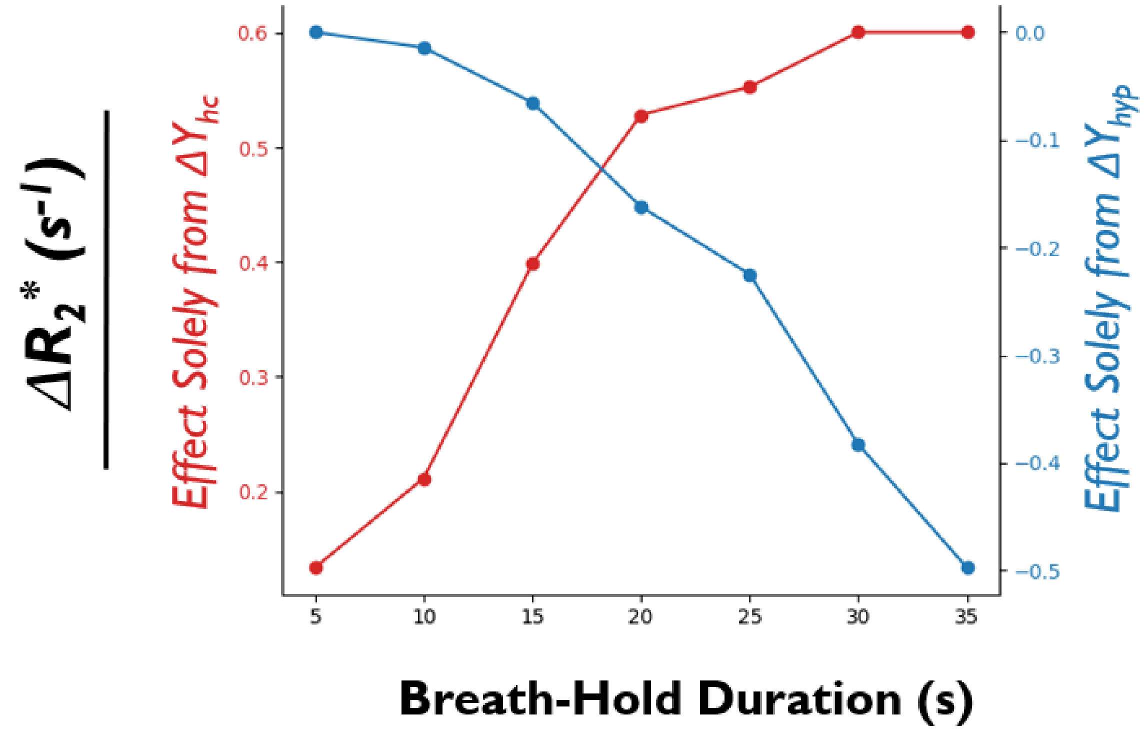

Supplementary Figure 2. Individual Effects from Hypoxia and Hypercapnia during Breath-Hold CVR_{BOLD}. *Refer to Supplementary Table 1 and Section 2.2 for simulation details and associated input values.*

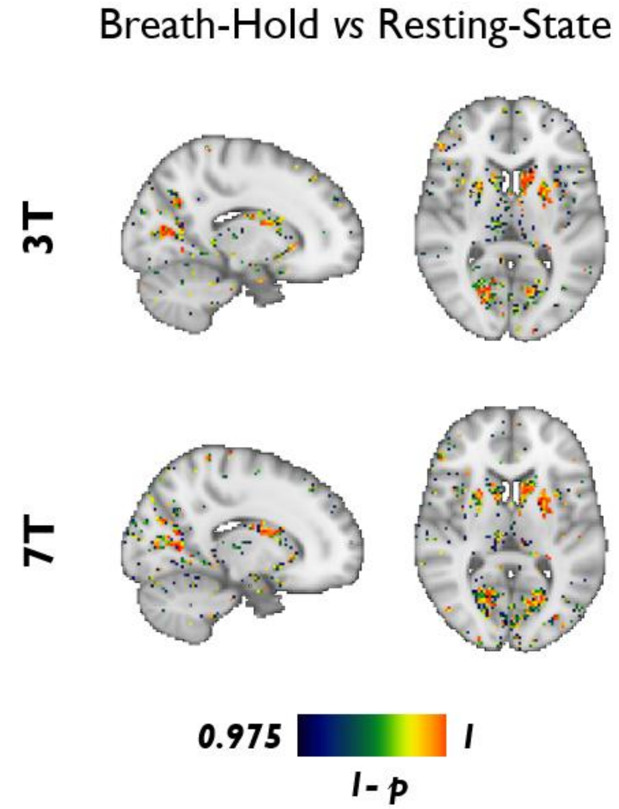

Supplementary Figure 3. Resting-State vs Breath-Hold CVR^* at 7 T and 3 T. *Statistical comparison (paired t-test, $\alpha = 0.05$, $n = 10$) in MNI152 2 mm anatomical space between breath-hold and resting-state CVR^* data at 3 T and 7 T. Only $p < 0.025$ displayed.*

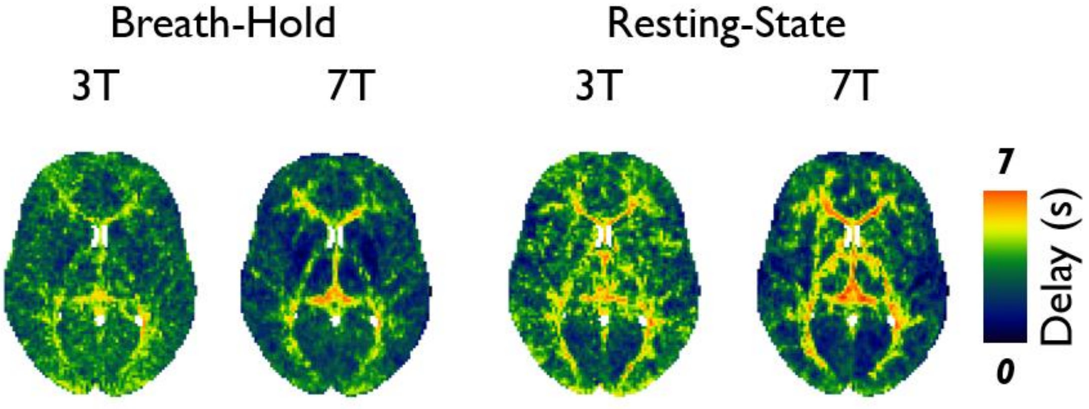

Supplementary Figure 4. Breath-Hold and Resting-State Delay Maps. *Subject-averaged delay (s) maps at both 3 T and 7 T in MNI152 2 mm anatomical space. Axial views are shown at the level of the putamen.*

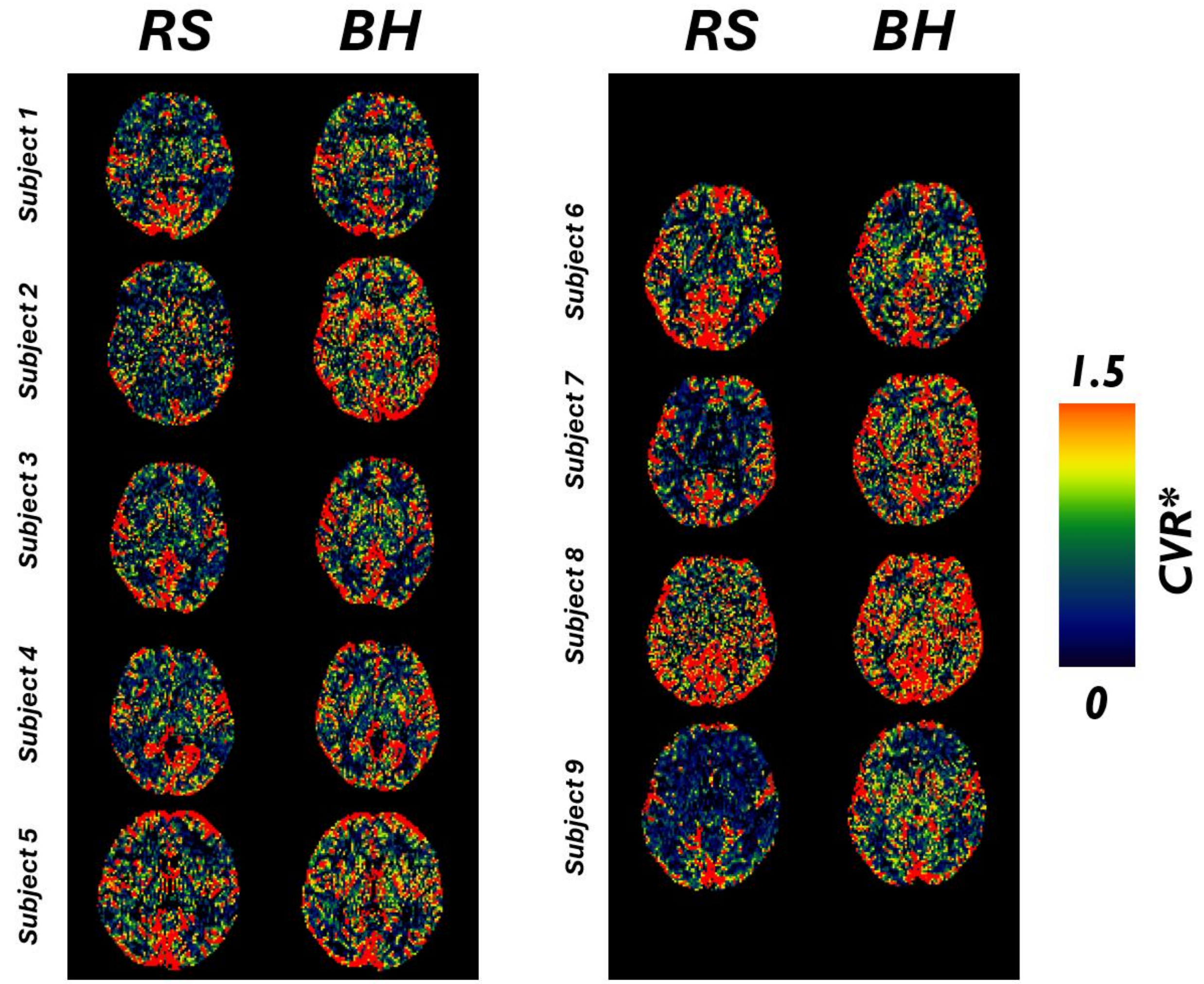

Supplementary Figure 5. Subject-wise CVR* Maps. *Resting-state and breath-hold maps are displayed for each subject at 7 T. Axial views are shown at the level of the putamen.*

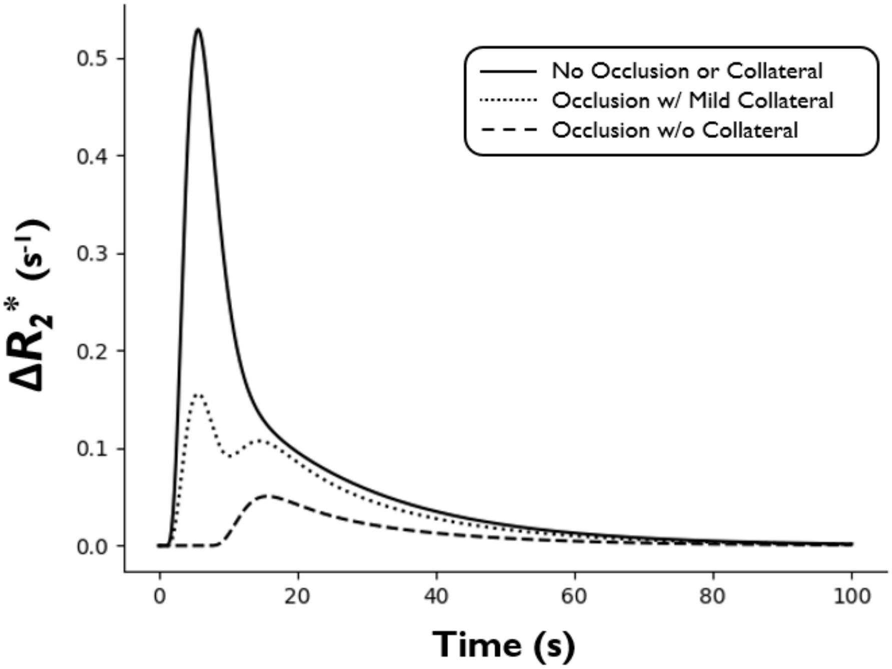

Supplementary Figure 6. Stenosis and Collateral Flow ΔR_2^* Tissue Time Courses. *Example ΔR_2^* tissue time courses from simulated tissue voxel corresponding to physiological scenarios with/without collateral flow and/or occlusion.*

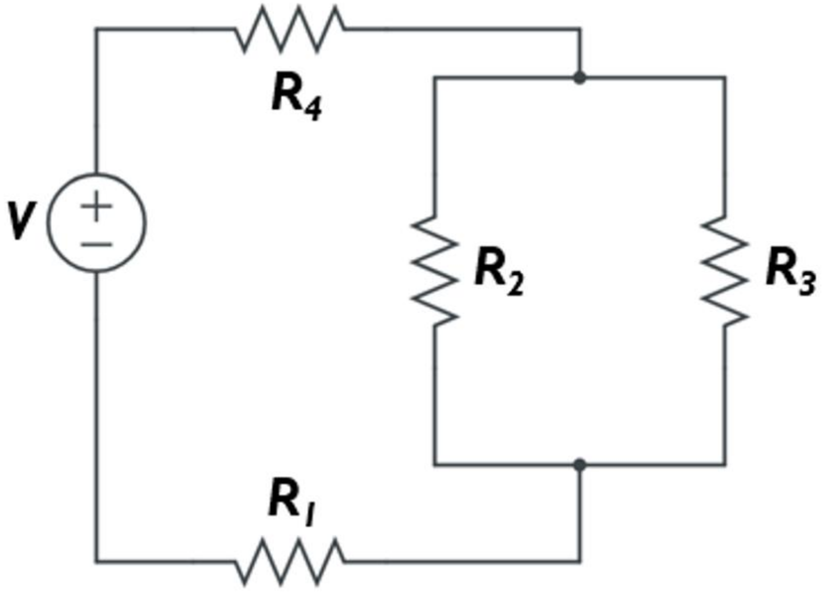

Supplementary Figure 7. Steal Physiology Circuit Schematic.

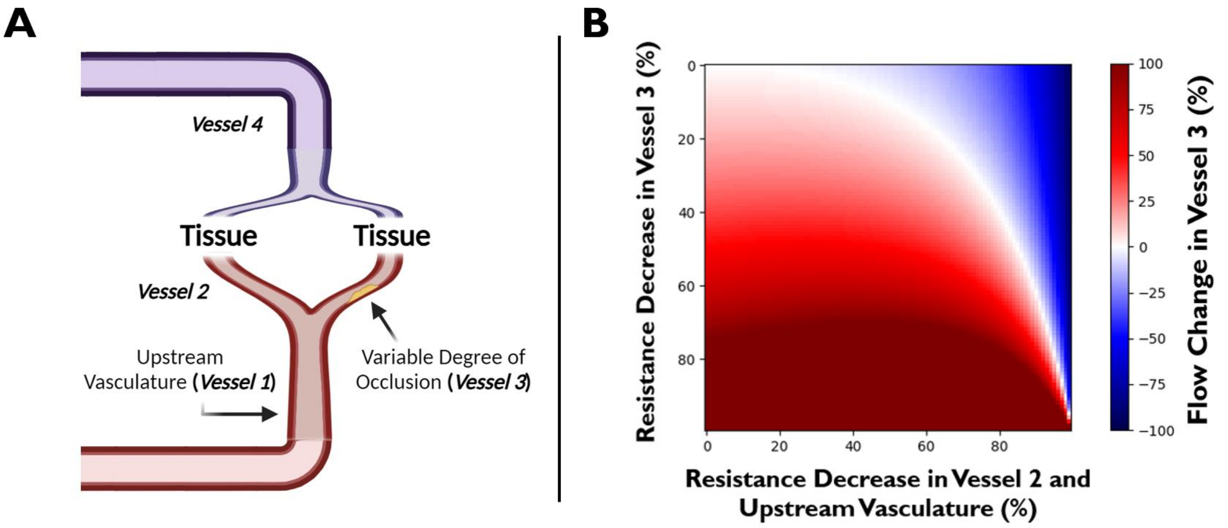

Supplementary Figure 8. Steal Physiology: Simulated Relationship Between Vascular Reactivity and Flow Change. A. *Schematic of steal physiology without upstream occlusion.* ***B.*** *Percentage flow change in Vessel 3 (in the presence of a vasodilative agent) as a function of vascular reactivity in Vessel 3 (y-axis) and vascular reactivity in Vessels 1+2 (x-axis).*

Supplementary Tables

**Breath-Hold Duration (s)**	ΔY_{hyp}	$\Delta P_{ET}CO_2$ **(mmHg)**	ΔCBF_p **(%)**	CVR_{CBF} **(%/mmHg)**	***CVR**BOLD* **Error (%)**
**5**	0	2.00	7.67	3.84	0
**10**	-0.0025	3.25	12.77	3.93	-5.60
**15**	-0.0115	6.50	27.12	4.17	-14.00
**20**	-0.0285	9.00	39.39	4.38	-26.40
**25**	-0.0395	9.50	41.95	4.42	-35.10
**30**	-0.0670	10.50	47.25	4.5	-54.30
**35**	-0.0869	10.50	47.25	4.5	-67.90

Supplementary Table 1. Simulated Effect of Hypoxia on CVRBOLD Estimation. ΔY_{hyp} *and* $\Delta P_{ET}CO_2$ *(relative to a* $P_{ET}CO_2$ *of 37 mmHg) obtained from Sasse et al., 1996.* ΔCBF_p *corresponding to* $\Delta P_{ET}CO_2$ *determined using relationships described in previous works (Grune et al., 2015; Poulin et al., 1996; Ramsay et al., 1993; Sato et al., 2012; Tancredi and Hoge, 2013).* CVR_{CBF} *calculated from* ΔCBF_p *and* $\Delta P_{ET}CO_2$*. CVRBOLD error represents the percent difference between the estimated CVRBOLD with vs without accompanying hypoxia (for a voxel with* CBV_0 *= 4% and* CVR_{Factor} *= 1).*
